# Allergenicity Assessment of Plant-Derived Sweet Proteins—In Silico, In Vitro, In Vivo, and Clinical Approach: A Systematic Review

**DOI:** 10.3390/molecules31091424

**Published:** 2026-04-25

**Authors:** Rima Hidayati, Puspo Edi Giriwono, Saraswati, Nuri Andarwulan, Dominika Średnicka-Tober

**Affiliations:** 1 Division of Food Science and Technology, Faculty of Engineering and Technology, IPB University, Kampus IPB Dramaga, Bogor 16680, Indonesia; rimahidayati@apps.ipb.ac.id (R.H.); pegiriwono@apps.ipb.ac.id (P.E.G.); saraswati-sa@apps.ipb.ac.id (S.); 2 Food Technology Study Program, Asa Indonesia University, East Jakarta 13620, Indonesia; 3 South-East Asia Food and Agricultural Science and Technology (SEAFAST) Center, IPB University, Bogor 16680, Indonesia; 4 Department of Functional and Organic Food, Institute of Human Nutrition Sciences, Warsaw University of Life Sciences, Nowoursynowska 159c, 02-776 Warsaw, Poland

**Keywords:** allergenicity, natural sweetener, non-communicable diseases, sweet proteins

## Abstract

Plant-derived sweet proteins are promising low-calorie natural sweeteners that may reduce dietary sugar intake and prevent non-communicable diseases. Although seven have been identified—thaumatin, miraculin, monellin, mabinlin, brazzein, pentadin, and curculin (neoculin)—only thaumatin is currently approved as a food additive. The development of others requires comprehensive safety assessments, particularly regarding allergenicity. This systematic review aims to investigate and synthesize allergenicity assessment methods (in silico, in vitro, in vivo, and clinical) applied to these seven sweet proteins. The literature searches were conducted following PRISMA guidelines across Scopus, PubMed, and Wiley Online Library databases, up to 30 November 2025, with no time restrictions. The risk of bias in selected studies was evaluated using GRADE. After the selection process, 14 out of 2634 studies met the inclusion criteria. Thaumatin, miraculin, monellin, and brazzein emerged as the most extensively studied proteins. In silico approaches (sequence and structural homology) and in vitro assays (digestibility and cell-based methods) were the most commonly employed methods. In contrast, in vivo studies (animal models) and clinical evaluations (skin prick tests, oral food challenges) were rarely reported. Allergenicity studies on pentadin, mabinlin, and curculin (neoculin) are limited, indicating a research gap that requires further study to support regulatory approval and consumer acceptance.

## 1. Introduction

Excessive sugar consumption has been reported to negatively impact health, notably by increasing the risk of diabetes [1]. According to the International Diabetes Federation, in 2021, an estimated 536.6 million adults aged 20–79 years worldwide were living with diabetes, and this number is projected to rise to 783.2 million by 2045. Of these cases, nearly 90% are classified as type 2 diabetes, indicating that the disease is largely preventable through the adoption of healthy lifestyle practices [2]. In addition to diabetes, excessive sugar intake has also been associated with an increased risk of other non-communicable diseases, including cardiovascular disorders [3], dental caries [4], and mental health issues [5]. However, reducing sugar in food products presents challenges, as sugar plays a significant role in determining food quality, including sweetness, flavor, and texture.

Sugar reduction in processed foods can be achieved through multiple strategies, including direct sugar reduction, multi-sensory integration, the use of non-nutritive sweeteners, and sweetness enhancers. While direct sugar reduction effectively lowers sugar content, it often compromises product palatability and may negatively impact consumer acceptance. Multi-sensory integration, however, aims to optimize sweetness perception by modifying sensory attributes such as color or aroma [6]. The use of sweeteners represents another strategy commonly implemented within the food industry [7]. Meanwhile, increasing health awareness among consumers has led to a preference for natural ingredients, clean labeling, and products with additional functional properties, without compromising taste. Consequently, the use of low-calorie and natural sweeteners presents a promising solution for meeting these consumer demands [8].

Among low-calorie and natural sweeteners, sweet proteins emerge as a promising option. As macromolecules, proteins also play a role in sensory attributes, including their ability to provide sweetness. Fruits of certain plants native to Africa or Asia can produce sweet-tasting proteins. With high sweetness intensity and low usage levels, sweet proteins represent a viable low-calorie sweetener alternative for consumers seeking to regulate blood sugar levels effectively. To date, seven sweet proteins extracted from fruits have been identified: thaumatin (*Thaumatococcus danielli*), monellin (*Dioscoreophyllum cumminsii*), miraculin (*Synsepalum dulcificum*), pentadin, brazzein (*Pentadiplandra brazzeana*), mabinlin (*Capparis masaikai*), and curculin/neoculin (*Curculigo latifolia*). Among these, only thaumatin has received approval as a food ingredient from the Scientific Committee for Food of the European Commission (SCF) and the Joint FAO/WHO Expert Committee on Food Additives (JECFA). The remaining sweet proteins require further research to confirm their safety and potential for application [6].

Although sweet proteins have traditionally been consumed by local communities in their native forms, their broader application as food ingredients or additives for mass consumers requires a comprehensive safety evaluation. In line with the European food legislation on novel foods (EU Regulation 2015/2283), such evaluation must comprehensively address nutritional, microbiological, toxicological, and allergenicity risks prior to market introduction in the EU [9]. Since the sweet taste is derived from proteins, these sweet proteins may pose a risk of inducing allergic responses in susceptible individuals. The prevalence of food allergies has risen globally among both children and adults in recent decades. While fruits are not part of the “Big Nine” allergens, certain fruits—including their peels, pulps, or seeds—can trigger allergic reactions in hypersensitive individuals. Fruit allergies may arise directly from the fruit itself, with or without an association with pollen sensitivities. Globally, the estimated prevalence of fruit allergies ranges from 0.029% to 8%, with the most commonly reported fruits causing allergies being banana, kiwi, avocado, mango, pineapple, and tomato [10]. In addition to these, some tropical or exotic fruits can cause region-specific allergies, even though information on these allergens remains limited and their characterization is often lacking. Examples include orange, mulberry, lychee, raspberry, pineapple, and sapodilla [11].

Given the rising prevalence of food allergies and the potential risks posed by specific food proteins, it becomes crucial to assess the allergenicity of sweet proteins, especially as they are increasingly explored as alternative sweeteners. A narrative review regarding the allergenicity of sweet-tasting proteins has been provided by Barre et al. [12]. Since that time, numerous studies have been published, but no systematic review has been conducted to comprehensively assess and synthesize the latest findings. Therefore, this paper aims to review recent findings regarding allergenicity assessment (in silico, in vitro, in vivo, and clinical studies) on seven known sweet proteins, providing insights into their safety for broader consumer use. Furthermore, beyond summarizing current findings, this review synthesizes the application of multiple allergenicity testing approaches to sweet proteins, establishing a methodological reference framework intended to support the allergenicity assessment of underexplored sweet proteins.

## 2. Results

**Figure 1:**
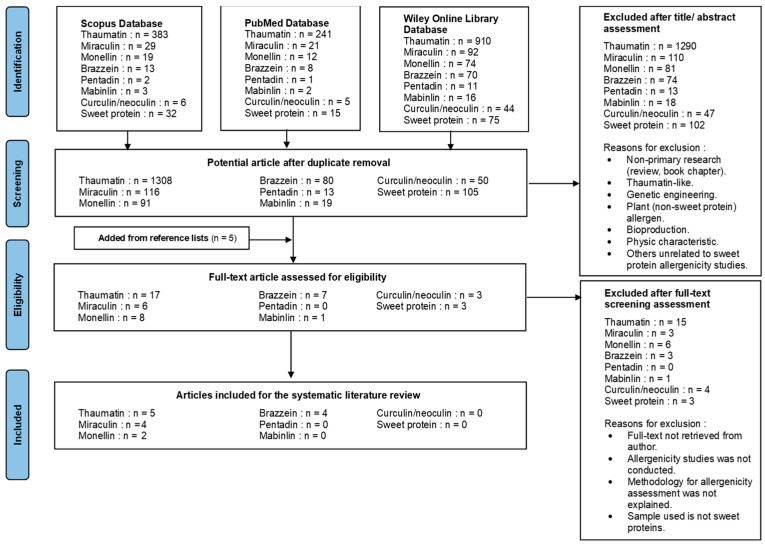
Flow chart of the selection of studies using the PRISMA method.

A systematic literature review regarding the allergenicity potential of the seven identified sweet proteins was conducted, as shown in Figure 1. A total of 2634 articles were identified from a search of three databases. Duplicate articles (455) were removed using Zotero. After screening the titles and abstracts of the remaining articles, 2120 articles were excluded because they represented non-primary research studies (reviews or book chapters) or discussed topics unrelated to allergenicity assessment of sweet proteins. A total of 51 articles were included for full-text assessment, and three additional articles from their reference lists were identified. In the next step, 32 articles were excluded because the full-text articles were not retrieved from the authors, the full texts did not report allergenicity studies, the methodology for allergenicity assessment was not explained, or the samples used were not sweet proteins. As a result, a total of 14 articles [13,14,15,16,17,18,19,20,21,22,23,24,25] were used for data analysis and synthesis. One article discussed both monellin and brazzein and was counted once in the total number of articles but included in both protein categories. The selected articles were published between 1983 and 2025 and were written in English. The list of articles included in this systematic literature review is presented in Table S1. This full-text assessment process led to the exclusion of 34 articles (Appendix A).

The results of the literature search indicate that in silico, in vitro, in vivo, and clinical approaches have been employed to assess the allergenicity of various sweet-tasting proteins. Based on these methods, a consolidated method for allergenicity assessment was developed, as presented in Table 1.

The risk of bias in articles selected in this study was evaluated using the GRADE tool, as presented in Table 2. The criteria evaluated included study limitations, inconsistency, indirectness, imprecision, and publication bias. Of the 14 selected articles, two studies were assessed as having low overall quality, four studies as having moderate overall quality, whereas the remaining studies were classified as high quality.

The article by Tafazoli et al. [14] was classified with serious imprecision because replicates were not reported in the in vitro digestibility assay. The corrigendum by Tafazoli et al. [14], which clarified the results of the original study, was scored with serious study limitations since the methods were not described in detail but only referred back to the previous article. The EFSA FAF [15] report was also considered to have study limitations, as the number of thaumatin samples used in the in vitro digestibility assay was not specified, despite the inclusion of whey protein and egg albumin as comparators. In addition, it was rated with imprecision because the number of replicates performed was not reported clearly. Some reports were non-peer-reviewed and submitted through EFSA’s call for data, which may result in publication bias.

The articles published by EFSA NDA et al. [20] present several methodological limitations, as they rely primarily on preliminary in silico analyses and internal ELISA screenings without clear reporting of dataset size, number of replicates, or methodological details, and some reports were applicant-submitted and non-peer-reviewed. Chung et al. [22] demonstrated a notable degree of indirectness, as their study did not directly assess the allergenicity of brazzein. Instead, they evaluated its anti-allergic potential by measuring the inhibitory effects on β-hexosaminidase release from rat basophilic leukemia (RBL-2H3) cells. Similarly, as observed in Tafazoli et al. [14], the in vitro digestibility study conducted by Lifshitz et al. [25] also did not report the number of replicates.

### 2.1. In Silico Approach in Sweet Protein Allergenicity Assessment

Antibodies can recognize allergenic proteins based on a specific amino-acid sequence (linear epitope) or on three-dimensional complexes (conformational epitope). This fundamental principle forms the basis of in silico methods used to predict the allergenicity of novel proteins rather than assess it directly. It constitutes bioinformatics-based analyses that utilize both the amino-acid sequence, which is publicly accessible via UniProt (https://www.uniprot.org/) [26], and the three-dimensional (3D) structure that can be retrieved from the Research Collaboratory for Structural Bioinformatics Protein Data Bank (RCSB PDB) (https://www.rcsb.org/) [27].

Based on the literature search, eight of the twelve selected articles reported some indications about the possible allergenicity of sweet proteins by using in silico methods [13,14,15,16,17,18,19]. Among the seven identified sweet proteins, only four (thaumatin, miraculin, monellin, and brazzein) had allergenicity that was already predicted by in silico methods. At the same time, no studies have been reported for pentadin, mabinlin, and curculin (neoculin). In silico methods used for allergenicity prediction of those four sweet proteins included sequence homology, structural homology, or in silico digestibility. The details of the in silico assessment results of each sweet protein are presented in Table 3.

#### 2.1.1. Sequence Homology

Sequence homology assessment is performed by comparing the target protein sequence with known allergenic protein sequences stored in a database, with this similarity subsequently defined as potential cross-reactivity. Currently, numerous allergen databases are available and freely accessible online, offering a diverse range of integrated features and analytical tools for the prediction of potential cross-reactivity [28]. Based on the systematic literature review, the most frequently used database/search tool (five out of eight articles) for sequence homology was AllergenOnline (http://allergenonline.com). Others were the National Center for Biotechnology Information (NCBI) Database (https://blast.ncbi.nlm.nih.gov), COMprehensive Protein Allergen Resource (COMPARE) (https://comparedatabase.org/), Structural Database of Allergenic Proteins (https://fermi.sdaponline.org) Allermatch (https://allermatch.org/), and Allerbase (https://bioinfo.unipune.ac.in/AllerBase/Home.html), all accessed on 10 December 2025. From the above-mentioned databases, AllergenOnline, COMPARE, SDAP, and Allermatch provide search tools for evaluating similarity level, including full-length sequence alignments, 80-amino-acid sequence alignments, and 6- or 8-amino-acid exact matches.

The sequence homology method has been employed to predict the potential cross-reactivity of four sweet proteins: thaumatin II (Uniprot ID: P02884), miraculin (Uniprot ID: P13087), monellin (Uniprot IDs: P02881 and P02882), and brazzein (Uniprot ID: P56522). Allergenicity prediction of thaumatin II using sequence homology was reported in a single study, which utilized full-length sequences, 80-mer, and 6–8-mer amino-acid alignments. Potential cross-reactivity was indicated by overall protein sequence identity >50%, 80-amino-acid sequence identity ≥ 35%, and the occurrence of ≥6 contiguous amino acids compared to known allergenic proteins. Based on sequence homology, thaumatin II was predicted to exhibit significant cross-reactivity with allergens from certain fruits (kiwi, apple, sweet cherry) and pollens (Arizona cypress, Mediterranean cypress, mountain cedar, temple juniper, and eastern red cedar) [13].

In contrast, sequence homology analysis of miraculin has been documented in three studies [14,15,16], employing various allergen databases including AllergenOnline, NCBI, and AllerBase. Cross-reactivity prediction between the miraculin sequence and known allergenic proteins was conducted using three consistent search models: full-length alignment, 80-mer sliding window, and exact 8-amino-acid match. Different from Baniulis et al. [13], the evaluation was based not only on percentage sequence identity but also on E-value. Full-length FASTA alignment using AllergenOnline version 19 predicted that miraculin has sequence similarity with a weak allergen (proteinase and aspartic protease inhibitors) from potato (*Solanum tuberosum*) and a strong allergen (trypsin inhibitors) from soy (*Glycine max*), although the sequence identity ranged only from 26.6% to 33.5%, with E-values between 6.4 × 10^−7^ and 6.4 × 10^−10^. BLAST alignment via NCBI revealed >50% sequence identities with allergens from peach, sesame, bitter lemon, tomato, latex, soy, and peanut, albeit with low query coverage. An 80-mer alignment also suggested possible reactivity between miraculin and allergens from potato and soybean, but no identical stretches of eight contiguous amino acids were identified [14]. Additionally, epitope mapping of miraculin against peanut allergens was conducted [16]. Overall, these analyses predicted that miraculin is unlikely to elicit significant cross-reactivity with known allergens above, since it does not meet the criteria for significant similarity between proteins.

Sequence similarity analysis of miraculin was conducted not only using the intact protein sequence but also through in silico-generated peptide fragments resulting from simulated pepsin digestion. Peptide fragments were retrieved from LC-MS/MS analysis. Alignment of these peptides using AllergenOnline, through both full-length sequence and 80-amino-acid segment comparisons, predicted that post-digestion miraculin peptides exhibited sequence identities ranging from 36% to 67% with several weakly allergenic proteins. However, no significant sequence homology to known allergens was identified based on the 80-amino-acid alignment criteria. These findings suggest that while partial similarity to low-potency allergens may exist, it is predicted that miraculin is unlikely to pose a substantial allergenic risk following gastric digestion [14].

In addition to aligning target protein sequences with known allergenic proteins using tools available in allergen databases, inter-protein sequence homology can also be evaluated through Receiver Operating Characteristic (ROC) curve analysis, which has been done in miraculin. In this context, the cut-off value obtained from the ROC curve is used to determine whether miraculin has the potential to elicit cross-reactivity with peanut allergens. The cut-off threshold was established by compiling data from allergens known to exhibit cross-reactivity with peanut (positive dataset) and those that do not (negative dataset). Sequence homology for each allergen pair in both datasets was quantified by multiplying the percentage identity (PI) by the percentage query coverage (PC). In addition to ROC analysis, the presence of peanut allergen epitopes in miraculin was examined using sequence alignment via NCBI BLASTp. Based on these approaches, miraculin was predicted to exhibit no significant cross-reactivity with peanut allergens [16].

Sequence homology analysis of monellin has been reported in only one study. Using full-length and 80-amino-acid sequence alignments via AllergenOnline and COMPARE databases, monellin is predicted to have no significant cross-reactivity with known allergens, because the percentage of sequence identity in both full-length and 80-amino-acid alignments did not meet the criteria. Beyond sequence-based assessments, Aller-CatPro 2.0 was utilized to predict allergenicity based on three-dimensional structural similarity. Consistent with the sequence alignment results, monellin showed no evidence of structural similarity to known gluten allergens [17].

For brazzein, two studies have predicted its allergenicity using in silico approaches. Similar to the allergenicity prediction conducted for thaumatin, miraculin, and monellin, brazzein’s amino-acid sequence was analyzed using AllergenOnline and Allermatch databases. Full-length sequence alignment revealed high identity between brazzein and a pathogenesis-related protein from peach, as well as with 20 putative allergens. However, further analysis using an 80-amino-acid sliding window and exact matches of eight contiguous amino acids did not identify any significant homology with known allergens. These findings suggest that while brazzein shares partial sequence identity with certain proteins, it is predicted not to elicit allergenic responses based on current in silico evidence [18,19].

#### 2.1.2. Structural Homology

Similar to sequence homology, structural homology involves comparing a target protein with allergenic proteins available in databases; however, the comparison is based on their three-dimensional (3D) structures rather than their amino-acid sequences. Structural homology assessment was conducted for thaumatin II, miraculin, and monellin. In thaumatin II, structural homology was employed to predict the spatial localization of potential allergenic epitopes, integrated with sequence and phylogenetic analyses. Multiple sequence alignment between thaumatin II and thaumatin-like proteins (TLPs) was initially performed using ClustalW v.1.83. Homologous sequences were subsequently organized into a cladogram using JalView v.2.3, from which protein clusters exhibiting sequence homology above 65% were selected. Antigenic region prediction was conducted using the EMBOSS Antigenic Server (http://liv.bmc.uu.se/cgi-bin/emboss/antigenic), which provides antigenic propensity scores for each predicted segment. Structural homology analysis was then carried out through comparative protein modeling using DeepView (Swiss-Pdb Viewer) v.3.7 and Swiss-Model to construct the three-dimensional structure of thaumatin II, which was not available at the time. Thaumatin I, with a known 3D structure, served as the modeling template. To determine whether the predicted antigenic regions were surface-exposed and thus accessible to antibodies, surface exposure assessment was performed using CCP4mg v.1.1.1 for structural visualization. Combined sequence and structural analyses identified six regions in thaumatin II that may function as allergenic epitopes [13].

In miraculin, structural homology analysis was conducted using a combination of computational tools, with allergenicity evaluation based on the statistical significance metrics provided by each software. Structural alignment was performed through three comparative approaches: (i) alignment of the miraculin protein structure with peanut allergen structures (Ara h 1, 2, 3, 5, 8, and 9); (ii) alignment of peanut allergen structures with bovine serum albumin as a negative control; and (iii) alignment of peanut allergen structures with known cross-reactive allergens as a positive control. Proteins were considered structurally homologous if they met specific thresholds across multiple platforms: a root-mean-square deviation (RMSD) value < 2.0 based on UCSF Chimera (https://www.cgl.ucsf.edu/chimera/, accessed on 10 December 2025), a *p*-value < 0.001 from FATCAT (https://fatcat.godziklab.org/), a Z-score > 2.0 from the DALI network server (http://ekhidna2.biocenter.helsinki.fi/dali/, accessed on 10 December 2025), and a TM-score between 0.5 and 1.0 from TM-align pairwise alignment. These criteria collectively provide a robust framework for predicting structural similarity and potential cross-reactivity between novel proteins and known allergens. Because miraculin failed to meet any of the four criteria for structural homology, it is predicted to have no significant structural homology with peanut allergens [16].

Unlike thaumatin II and miraculin, structural homology analysis of monellin was conducted using AllerCatPro 2.0 (https://allercatpro.bii.a-star.edu.sg/), a web-based platform designed to predict protein allergenicity. In addition to evaluating structural similarity, AllerCatPro 2.0 also predicts potential cross-reactivity based on sequence homology using a linear window approach. Evaluation using AllerCatPro predicted that monellin had no evidence of allergenicity (E-value 0.001) and no evidence of similarity to known gluten allergens [20].

#### 2.1.3. In Silico Digestibility

The reliability of cross-reactivity prediction through sequence and structural homology is highly dependent on the availability and completeness of allergen sequence data within reference databases [29]. In addition to database-driven comparisons, another in silico strategy for evaluating the allergenic potential of novel proteins involves assessing their stability against digestive enzymes. This approach is based on the observation that proteins resistant to proteolytic degradation are more likely to elicit immune responses [30], whereas hydrolyzed proteins typically exhibit reduced allergenicity [31,32]. To simulate protease susceptibility, protein sequences obtained from UniProt can be analyzed using the Expasy PeptideCutter web server (https://web.expasy.org/peptide_cutter/). This tool provides detailed output, including the number and positions of cleavage sites, resulting peptide sequences, lengths, and molecular masses [33]. Among the seven identified sweet proteins, monellin is the only one that has undergone in silico digestibility analysis using pepsin and trypsin enzymes. Freeman et al. [17] reported that monellin possesses 15 cleavage sites for each enzyme, resulting in an average peptide fragment size of 3.43 amino acids.

### 2.2. In Vitro Approach in Sweet Protein Allergenicity Assessment

Various in vitro methods have been developed to examine the immune mechanisms involved in IgE-mediated food allergies, both for diagnostic and research purposes. In vitro detection and quantification of allergens in food products can be performed using DNA-based immunochemical methods, separation techniques, peptide marker assays, and assays for the release of allergy mediators (such as histamine and/or β-hexosaminidase) [34].

The systematic literature review identified six out of twelve selected articles that assessed the allergenicity of sweet proteins using in vitro methods. Similar to in silico allergenicity prediction, only four (thaumatin, miraculin, monellin, and brazzein) have already assessed their allergenicity potential by in vitro, and no studies have been reported for pentadin, mabinlin, and curculin (neoculin). In vitro methods used for assessing the potential allergenicity of those four sweet proteins were cell-based methods, in vitro digestibility tests, or indirect ELISA, as presented in Table 4.

#### 2.2.1. Cell-Based Method

A cell-based method for assessing the potential allergenicity of proteins is based on the mechanisms of food allergic reactions. A protein can trigger an allergic reaction if it can cross-link with specific IgE antibodies present on the surface of effector cells, such as basophils and mast cells. This binding can trigger the release of allergic mediators, such as histamine, leukotriene, or β-hexosaminidase. The increase in the allergic mediator response can then be measured, thus evaluating its potential as an allergen. However, this response measurement can only be performed if the cells used have passed the sensitization stage [35]. In vitro potential allergenicity assessment of sweet proteins using cell-based methods has been conducted with mast cells and basophil cells. These cells are used to measure the response of allergic mediators following stimulation by sweet proteins, namely histamine or β-hexosaminidase. In addition to measuring the concentration of allergic mediators, the expression of the basophil cell degranulation marker (CD63) and the number of degranulated mast cells are also measured [21,22,23,24].

Rat mast cells and basophils have been employed as in vitro models to evaluate the allergenic potential of thaumatin and brazzein. In the case of thaumatin, histamine release from rat mast cells was quantified using fluorimetric analysis following protein stimulation. The results demonstrated that the thaumatin extract, obtained from the arils of *T. daniellii* through highly selective ultrafiltration, required a higher concentration to induce 50% histamine release compared to Synacthen, indicating a lower allergenic potential [21].

Additionally, indirect assessments of mast cell degranulation in guinea pigs were conducted to evaluate the allergenicity potential of brazzein and monellin. Indirect assessment was done by measuring the percentage of degranulated mast cells obtained from guinea pig blood sera after sensitization with the sweet protein by using a microscope. Microscopic observations revealed that the percentage of degranulated mast cells following stimulation with brazzein or monellin did not differ significantly from those stimulated with distilled water, suggesting minimal allergenic potential [24].

Basophil activation assays have been employed to evaluate the allergenic potential of thaumatin and brazzein. In the case of thaumatin, flow cytometry was utilized to measure CD63 marker expression following stimulation with a thaumatin–gum arabic powder mixture. Basophils were isolated from whole blood samples of individuals with upper respiratory allergies, both symptomatic and asymptomatic. The results indicated that all symptomatic individuals exhibited elevated CD63 expression, whereas no activation was observed in basophils from asymptomatic individuals [23].

Rat basophilic leukemia (RBL-2H3) cells were also used as an in vitro model to assess β-hexosaminidase release, a marker of basophil degranulation, in brazzein. Spectrophotometric analysis revealed that brazzein inhibited approximately 27% of β-hexosaminidase release, suggesting potential anti-allergic properties [22]. Collectively, these findings predicted that thaumatin may elicit allergic responses in sensitized individuals, albeit requiring relatively high concentrations to do so. In contrast, both basophil-based assays predicted that brazzein does not exhibit allergenic potential under the tested conditions.

#### 2.2.2. In Vitro Digestibility Test

Protein digestibility testing has long been incorporated into potential allergenicity assessment protocols, beginning with the pepsin resistance assay introduced in 1996. This assay evaluates protein stability under gastric conditions, primarily focusing on susceptibility to pepsin-mediated hydrolysis. Over time, more physiologically relevant models have been developed, including sequential digestion simulations that mimic both gastric and intestinal environments. These advanced protocols involve initial exposure to pepsin under acidic conditions, followed by treatment with pancreatic enzymes and bile salts to simulate intestinal digestion [31,36].

Based on the systematic literature review, miraculin, thaumatin, monellin, and brazzein are sweet proteins that have been reported to have undergone in vitro digestibility assays [14,15,17,19]. The digestion simulation model applied to thaumatin, brazzein, and monellin employed a two-phase digestion process, involving sequential enzymatic hydrolysis by pepsin and pancreatin. Meanwhile, miraculin has only been tested by pepsin digestion. The potential allergenicity was evaluated by comparing the intensity of the target protein bands from SDS-PAGE analysis before and after simulated digestion. A reduction in band intensity following digestion indicates that the protein is readily hydrolyzed. Proteins resistant to digestive enzymes are generally more effective in eliciting immune responses [30], whereas hydrolyzed proteins typically exhibit a lower risk of allergenicity [31,32].

In vitro digestibility analysis of thaumatin revealed that its digestibility score was similar to that of whey protein and egg albumin. SDS-PAGE results further demonstrated that thaumatin was readily hydrolyzed by pepsin and pancreatin enzymes [15]. At the same time, brazzein is resistant to gastric enzymes (pepsin) but is partially digested by intestinal enzymes. During simulated gastric digestion, brazzein powder (140 ppm; produced by Sweegen) exhibited no detectable hydrolysis by pepsin, indicating resistance to enzymatic degradation under gastric conditions. These results are based on the intensity of the brazzein band, which remained stable until the 120th minute of the gastric digestion simulation. Subsequently, the intensity of the brazzein band after passing through intestinal digestion decreased slightly with increasing simulation time [19]. Another sweet protein, monellin, showed instability during gastrointestinal digestion. A study by Freeman et al. [14] showed that the intensity of the monellin band began to decrease after 30 min of gastric digestion, then decreased significantly (>90%) after passing through intestinal digestion [17]. Based on band intensity analysis, the results of simulated two-phase digestion indicated that both brazzein and monellin exhibit high stability when exposed to gastric enzymes but are subsequently hydrolyzed by intestinal enzymes.

Unlike brazzein and monellin, the in vitro digestion simulation of miraculin (0.1 mg/mL) was conducted exclusively using pepsin (5.45 U/µg) in simulated gastric fluid (SGF). Following incubation at 37 °C for 20, 40, and 60 min, the digestion products were analyzed by SDS-PAGE and LC-MS/MS. SDS-PAGE results revealed that miraculin was completely digested after 20 min, as evidenced by the absence of protein bands from that time point onward. In parallel, LC-MS/MS analysis indicated that miraculin yielded 61 unique peptides after 10 min of digestion, suggesting that the protein is highly susceptible to proteolytic degradation and is unlikely to remain intact for absorption in the gastrointestinal tract. Collectively, these findings suggest that miraculin exhibits a very low allergenic potential [14].

#### 2.2.3. Indirect ELISA

Enzyme-linked immunosorbent assay (ELISA) is an immunological technique widely used to semi-quantitatively detect or measure specific components—such as antibodies (IgE), antigens, or other proteins—in biological samples (e.g., serum, plasma, urine). In allergenicity studies, ELISA relies on the specific interaction between an antigen (allergen) and an enzyme-conjugated antibody. Upon binding, the enzyme catalyzes a colorimetric reaction with a substrate, producing a measurable color change. The absorbance is then quantified using a spectrophotometer to determine the presence and concentration of the target component [34,37].

Based on the systematic literature review, miraculin is the only sweet-tasting protein that has undergone potential allergenicity assessment using the indirect ELISA method. Serum from individuals with peanut allergy was employed in this evaluation, as commercial ELISA testing indicated the potential presence of peanut allergens or structural similarity between peanut proteins and those found in miracle berry. The results showed that miraculin, in the form of lyophilized miracle berry extract, had no significant difference in absorbance compared to the negative control (bovine serum albumin), while being significantly lower than the peanut protein extract [16].

### 2.3. In Vivo Approach in Sweet Protein Allergenicity Assessment

In vivo allergenicity testing is performed using animal models by administering allergens via oral, respiratory, or dermal routes to evaluate immune responses and clinical manifestations. Only two out of twelve identified articles assessed the allergenicity of sweet proteins using in vivo methods, and they focused only on thaumatin, monellin, and brazzein. Table 5 shows the sample preparation, in vivo model, treatment, method, allergenicity determination criteria, and results of in vivo allergenicity assessment of those three sweet proteins.

The animal models used in sweet protein studies were guinea pigs, rats, and baboons. To determine their allergenicity, the methods used were intramuscular injection to observe the ileum contraction response, and a combination of subcutaneous, intradermal, and intravenous injections to observe passive cutaneous anaphylaxis (blueing response). Additionally, organ responses (skin, conjunctiva, and nose) were also used to determine allergenicity after exposure to sweet proteins by intragastric administration.

#### 2.3.1. Animal Models

Intramuscular injection involves administering allergens into a specifically selected muscle. Due to the high vascularity of muscle tissue, the injected substance can rapidly enter systemic circulation and reach target areas [38]. Among the seven known sweet-tasting proteins, this method has only been applied to assess the allergenicity of thaumatin, using the Schultz–Dale method. This technique enables the study of allergic reactions (anaphylaxis) by employing previously sensitized smooth muscle tissue, which is subsequently challenged with specific allergens [39]. In the case of thaumatin, thaumatin extract was intramuscularly injected into the ileum muscle of guinea pigs to induce the formation of specific antibodies against the protein. After a sensitization period of 12–30 days, the animals were sacrificed, and the ileum segments were excised. At this stage, the smooth muscle tissue contained mast cells bound to allergen-specific IgE antibodies. The sensitized ileum segments were mounted in a Schultz–Dale bath and subsequently challenged with either thaumatin or egg albumen. The contractile responses of the ileum were recorded and compared with those elicited by egg albumen [21].

To determine the concentration of anaphylactic antibodies in male rat serum using the passive cutaneous anaphylaxis (PCA) titration method, three types of injections, subcutaneous, intradermal, and intravenous, are administered across two main stages. In the first stage, initial sensitization is achieved through subcutaneous injection to induce an allergic response in donor rats. After 11–12 days, the donor rats are euthanized, and serum containing anaphylactic antibodies is collected. In the second stage, the donor serum is injected intradermally into recipient rats to transfer the anaphylactic antibodies to the skin. After 48 h, an intravenous injection is administered to the recipient rats to trigger an anaphylactic reaction and enable result visualization [21]. A higher PCA titer (i.e., greater dilution) indicates a higher concentration of anaphylactic antibodies in the serum. Compared to egg albumen, thaumatin exhibits a lower PCA titer, suggesting that the concentration of anaphylactic antibodies induced by thaumatin is lower than that induced by egg albumen. Based on both in vivo assays, thaumatin demonstrates allergenic potential, although its potency appears comparable to or lower than that of egg albumen.

#### 2.3.2. Intragastric Administration

In contrast to thaumatin, in vivo allergenicity testing of monellin and brazzein was conducted using guinea pig models. The sensitization phase involved daily intragastric administration of brazzein or monellin for 21 consecutive days. The administered dose for each group corresponded to the sweetness level equivalent to sucrose, referred to as the equivalent dose (ED). In guinea pigs, sucrose elicits a sweet taste at a dose of 4350 mg/kg body weight, whereas brazzein and monellin require only 2.17 mg/kg and 1.45 mg/kg body weight, respectively, to achieve comparable sweetness (1× EDsucrose standard).

Allergenicity testing was performed not only at 1× ED sucrose standard but also at 10× EDsucrose standard. Ten to twelve days after the sensitization phase, sweet protein extracts were applied to the surface of the skin, conjunctiva, and nasal mucosa, and visual allergic responses were observed. Cutaneous allergic responses were indicated by changes in skin coloration (hyperemia) or increased skin fold thickness. Conjunctival responses were assessed based on vascular patterns and the presence of hyperemia. Nasal responses were characterized by hyperemia, sneezing, edema, or increased mucus secretion. The study demonstrated that, at the tested doses, neither brazzein nor monellin elicited allergic responses in guinea pigs [24].

### 2.4. Clinical Test Approach in Sweet Protein Allergenicity Assessment

Clinical allergenicity testing involves direct exposure of the test protein to human subjects, either through skin prick testing or oral administration (oral food challenge). Among the seven identified sweet-tasting proteins, thaumatin is the only one that has undergone clinical allergenicity testing. Table 6 summarizes the sample preparation, subjects, treatment protocols, testing methods, allergenicity determination criteria, and results of thaumatin’s clinical assessment.

#### 2.4.1. Skin Prick Test

The first clinical study was conducted by Higginbotham et al. [21] involving 140 laboratory and pilot plant employees who had been exposed to thaumatin over a seven-year period. The tested samples included commercial thaumatin, purified thaumatin, mixtures of thaumatin with gum arabic (freeze-dried or spray-dried), and non-thaumatin plant constituents. Positive reactions were determined based on the diameter of the wheal produced. Skin prick testing was performed using a dose equivalent to 10,000 PNU/mL of purified thaumatin. The results showed that 12 atopic individuals and one non-atopic individual exhibited positive reactions to thaumatin. Furthermore, the addition of gum arabic to thaumatin did not reduce its allergenicity.

Skin prick testing on thaumatin was also reported by Tschannen et al. [23], following a report received in 2014 by the Swiss Occupational Health Department regarding four workers exhibiting upper respiratory symptoms at a chewing gum factory utilizing a mixture of thaumatin powder and gum arabic as ingredients. Of the eight workers investigated, four were frequently exposed to the thaumatin–gum arabic mixture, specifically those responsible for ingredient addition during production. All workers underwent skin prick testing using ten common commercial allergens, purified thaumatin, purified gum arabic, guar gum, and locust bean gum. The findings revealed that four symptomatic workers (presenting with rhinorrhea, nasal obstruction, and sneezing) did not report any oral or gastrointestinal symptoms upon chewing gum consumption. Skin prick tests in workers with rhinitis symptoms showed strong positive reactions to thaumatin powder.

Skin prick test results also revealed that three symptomatic workers exhibited positive reactions to common allergens, suggesting that genetic predisposition (atopy) may contribute to allergic responses to thaumatin and gum arabic. In contrast, asymptomatic workers showed negative responses in skin prick tests to both purified thaumatin and gum arabic. However, after the factory replaced powdered thaumatin with its liquid form, symptom reduction was observed in two workers who had previously tested positive for gum arabic-specific IgE and skin prick reactivity. Based on the investigation, thaumatin was identified as the primary trigger of allergic reactions, particularly affecting the upper respiratory tract [23].

#### 2.4.2. Oral Food Challenge

In addition to the skin prick test, Higginbotham et al. [21] also reported an oral food challenge conducted to assess the allergenicity of thaumatin. The study involved 10 volunteers who were instructed to consume 100 mg of thaumatin or lactose (placebo) encapsulated in gelatin daily for 14 days each, following a double-blind crossover design. The observed responses included skin prick test results, serum IgE levels, and passive cutaneous anaphylaxis (PCA) assays performed on baboons or rhesus monkeys. The clinical trial results indicated that none of the three tests yielded positive allergic reactions.

Based on the results of the systematic literature review, the allergenicity profiles of each sweet protein are summarized in Table 7. Thaumatin is the only sweet protein that has undergone a comprehensive allergenicity evaluation, ranging from in silico prediction to clinical assessment. Based on the methods applied, thaumatin shows potential as a respiratory allergen due to its cross-reactivity with certain pollens and fruits. However, no cases of allergic reactions have been reported following its consumption. Brazzein and monellin’s potential allergenicity has been assessed up to the in vivo level. Meanwhile, potential allergenicity testing of miraculin has been limited to in vitro studies. However, further experimental and clinical investigations are needed to confirm these predictions. No allergenicity studies are currently available for pentadin, mabinlin, and curculin (neoculin).

## 3. Discussion

### 3.1. In Silico Approach in Sweet Protein Allergenicity Assessment

#### 3.1.1. Sequence Homology

The systematic literature review identified three in silico approaches commonly employed to assess the allergenicity of sweet proteins: sequence homology, structural homology, and in silico digestibility. In the sequence homology method, potential cross-reactivity is determined based on the level of similarity between the sweet protein sequence and known allergenic proteins archived in allergen databases. This similarity is assessed through full-length sequence alignments, 80-amino-acid segment alignments, and exact matches of 6- or 8-amino-acid contiguous sequences. Although there were three methods to evaluate potential cross-reactivity using bioinformatic tools, the results of 80-amino-acid alignment with greater than 35% identity are considered more meaningful than full-length sequence comparisons or 6–8 amino-acid exact matches. This approach is recommended by the FAO/WHO and has been widely adopted in allergenicity assessments. This threshold is considered a conservative approach, given that cross-reactivity more commonly occurs when sequence similarity exceeds 50–70% [40].

Full-length sequence alignment searches compare the entire length of the target protein with the complete sequence of a known allergen. In AllergenOnline searches, similarity between protein sequences in full-length alignment is evaluated based on two parameters: the E-value and percent identity (% identity). The E-value (expectation value) is a statistical measure that indicates how likely sequence similarities are to occur by chance. A smaller E-value indicates a greater degree of similarity between proteins. This value is influenced by the length of the alignment, the percent identity and similarity, and the size of the database. In addition to the E-value, the potential for cross-reactivity is also evaluated by percent identity. A protein has the potential to trigger an allergic response if it has a percentage identity of >70% with a known allergenic protein. This is because, although IgE epitopes consist of only short amino-acid sequences, to mediate an allergic reaction, at least two different IgE epitopes are needed in a single protein, which are often in a conformational form. Therefore, cross-reactive allergenicity between proteins may occur when the resulting E-value is extremely low (e.g., ≤10^−7^) and the sequence identity exceeds 70% [41].

Evaluation of full-length FASTA sequence alignment for thaumatin II and monellin using AllergenOnline was solely based on sequence identity percentage (>50%), with the E-value neither reported nor considered in assessing potential cross-reactivity. In contrast to thaumatin, although also utilizing AllergenOnline, the in silico potential allergenicity assessment of miraculin incorporated both sequence identity percentage and E-value. Although full-length sequence alignment tends to produce fewer false positives, this approach may overlook shorter regions of significant similarity that are biologically relevant. Moreover, the outcome of full-length alignment is highly dependent on the E-value, which can vary across databases and may not consistently reflect functional similarities [42,43,44,45].

The 80-amino-acid alignment search utilized a sliding window method, where sequential 80-residue segments (e.g., positions 1–80, 2–81, 3–82, and so forth) were compared sequentially across each complete sequence. Subsequently, each subsequence was subjected to alignment against the sequence in the reference database. Meanwhile, the 6- or 8-amino-acid exact match search can identify an identical match of six or eight consecutive residues within the complete sequence of a known allergen. This search employs a word match algorithm to find perfect identity across a specified number of contiguous amino acids between the target protein sequence and known allergenic protein sequences within the reference database [46]. Meanwhile, the results of 6-mer word exact matches are not considered a primary criterion in the evaluation of cross-reactivity due to the high likelihood of false positives and the fact that short sequence matches do not adequately reflect overall similarity [29,47,48].

As previously mentioned, the 80-amino-acid sliding window alignment is a primary criterion used in bioinformatic tools to evaluate cross-reactivity between proteins. The alignment results for thaumatin II revealed potential cross-reactivity not only with various fruits and vegetables (kiwi, chili pepper, tomato, apple, and sweet cherry) but also showed sequence identity ≥ 35% with several pollen sources, including Arizona cypress, Mediterranean cypress, mountain cedar, temple juniper, and eastern red cedar [13]. Although not classified among the “Big 9” allergens, these plant-derived substances may exert adverse effects on sensitive individuals [49].

Allergens in fruit can cause oral allergy syndrome (OAS). Kiwi (*Actinidia deliciosa*) is recognized as a highly allergenic fruit, with Act c 2 identified as a major allergen. A common clinical manifestation is OAS, characterized by itching and swelling in the oral cavity and throat [50]. Chili pepper allergenicity has been predicted through bioinformatic analyses [51] and reported in cases of occupational exposure among spice mill workers [52], both of which have been associated with the onset of OAS. Tomatoes have been reported to have cross-reactivity with Japanese cedar pollen, potentially triggering OAS in sensitized individuals [53]. Additionally, an allergen derived from Japanese cedar (*Cryptomeria japonica*) pollen has been implicated in allergic rhinitis, with symptoms including sneezing and itchy and watery eyes [54,55]. Allergens from pollen that showed cross-reactivity with thaumatin, are Arizona cypress (Cup a 3), Japanese cedar (Cry j 3), Mediterranean cypress (Cup s 3), mountain cedar (Jun a 3), temple juniper (Jun r 3), and eastern red cedar (Jun v 3). This group of allergens can cause respiratory allergies (allergic rhinitis, conjunctivitis, and asthma [56,57,58,59,60]. Because of it has cross-reactivity with these fruits and pollen, thaumatin II has the potential to cause oral allergy syndrome or rhinitic allergy in sensitive individuals.

Based on the 80-amino-acid sequence alignment, miraculin exhibited seven matches (identity 35.9–39.3%) with allergenic proteins from soybean (proteinase inhibitor and aspartic protease inhibitor 11) and potato Kunitz trypsin inhibitor and trypsin inhibitor subtypes A and B). These proteins are not classified as major allergens. The primary allergenic proteins in soybean are glycinin and the β-conglycinin α-chain [61]. Although uncommon, potato allergy has been reported, with clinical manifestations including urticaria, gastrointestinal disturbances, and anaphylaxis. Potato allergy has also been associated with pollen sensitization [62]. Patatin (Sol t 1) is recognized as the major allergen in potato and other Solanaceae species. While protease inhibitors may contribute to allergic reactions to potato, their allergenic potential is considered lower than that of patatin [63]. Meanwhile, following pepsin digestion, no sequence homology was observed between peptide fragments of miraculin and known allergens listed in the AllergenOnline database [14], suggesting that miraculin does not pose a risk of triggering allergic reactions. Other sweet proteins, monellin and brazzein, showed no sequence identity (≥35%) with any known allergens in the AllergenOnline database.

#### 3.1.2. Structural Homology

As previously noted, antibodies can recognize allergenic proteins through three-dimensional conformational epitopes, making structural homology analysis a critical component in evaluating the potential cross-reactivity of novel proteins with known allergens. Several computational tools are available to perform structural homology assessments, many of which are integrated with protein sequence and structure databases. Findings from the systematic literature review indicate that only three sweet proteins—thaumatin II, miraculin, and monellin—have been evaluated for allergenicity using in silico structural homology approaches. These structural analyses were conducted in conjunction with sequence homology evaluations, highlighting the complementary nature of both methods. Protein structures used for homology analysis were obtained either from the Protein Data Bank (https://www.rcsb.org/) or generated through homology modeling using tools such as Swiss-Model. Structural homology assessments provide valuable insights into potential immunogenicity and are essential for the comprehensive safety evaluation of novel food proteins.

Among the seven known sweet-tasting proteins, thaumatin, miraculin, and monellin have been analyzed for their allergenicity based on structural characteristics. Homology determination among protein structures can be performed using one or a combination of bioinformatics tools. In the case of thaumatin, the analysis employed an integrated approach involving ClustalW for multiple sequence alignment, the EMBOSS Antigenic Server for antigenic region prediction, DeepView and Swiss-Model for three-dimensional structure construction, and CCP4mg for structural visualization. Meanwhile, the assessment of miraculin employed a combination of tools, including UCSF Chimera, FATCAT, the DALI network server, and TM-score.

UCSF Chimera is a molecular visualization and analysis software capable of comparing protein structures and performing sequence-based alignments. Structural homology between two proteins in UCSF Chimera is quantified using the root-mean-square deviation (RMSD) value, which represents the spatial deviation of α-carbon atoms between paired residues. RMSD measures the atomic distance after optimal superposition of the two structures, with lower RMSD values indicating greater structural similarity [64]. Comparable structural comparisons can also be performed using the FATCAT (Flexible structure Alignment by Chaining Aligned fragment pairs allowing Twists) web server [65]. Unlike RMSD, FATCAT evaluates structural homology based on the statistical significance of the alignment, expressed as a *p*-value. The *p*-value reflects the probability of obtaining equal or greater similarity by chance between two randomly selected structures; thus, a lower *p*-value denotes a more statistically significant similarity [66]. In contrast, the DALI (Distance-matrix ALIgnment) web server assesses structural homology using a Z-score, which indicates the likelihood of shared evolutionary ancestry between proteins. A Z-score greater than 20 is generally considered indicative of homologous relationships [67]. These complementary tools provide robust frameworks for evaluating structural similarity and potential cross-reactivity in allergenicity assessments.

Unlike thaumatin II and miraculin, structural homology analysis of monellin was conducted using one tool, namely AllerCatPro 2.0 (https://allercatpro.bii.a-star.edu.sg/). This platform integrates allergenic protein data from multiple authoritative sources, including the World Health Organization/International Union of Immunological Societies (WHO/IUIS), COMPARE, AllergenOnline, UniProtKB, and Allergome. The output generated by AllerCatPro 2.0 provides a direct classification of the allergenic potential of a query protein, indicating whether there is strong, weak, or no evidence of cross-reactivity with known allergens in the database. This integrated approach enables rapid and comprehensive screening of novel proteins for potential allergenicity [68].

In addition to the tools previously mentioned, another resource available for evaluating protein structures in relation to allergenicity is the Structural Database of Allergenic Proteins (SDAP 2.0) (https://fermi.sdaponline.org/). Beyond identifying peptides with sequence homology and physicochemical similarity to known linear IgE-binding epitopes, the latest version of SDAP also enables the prediction of conformational IgE epitopes based on the three-dimensional structure of proteins [69].

#### 3.1.3. In Silico Digestibility

In silico digestibility analysis provides insights into the stability of target proteins against digestive enzymes, utilizing protein sequence as input data. One of the tools commonly used for this analysis is PeptideCutter. The results of in silico digestion using this tool provide information on the number of cleavage fragments, which reflects the structural stability of the protein. A high number of cleavage fragments may indicate low structural integrity, suggesting a reduced likelihood of allergenic potential due to increased digestibility [70].

In sweet proteins, in silico digestibility analysis using PeptideCutter has only been performed on monellin [17]. It was reported that monellin possesses 15 cleavage sites for each digestive enzyme, resulting in an average peptide fragment size of 3.43 amino acids. These findings indicate that monellin is highly susceptible to enzymatic digestion in silico, suggesting a low potential for allergenicity. This can be explained by the general consensus that proteins or protein fragments of approximately 3.5 kDa are sufficient to enable cross-linking of at least two IgE epitopes, thereby posing a risk of provoking IgE-mediated allergic reactions [65]. Meanwhile, a study conducted by Tanabe [71] demonstrated that epitopes consisting of at least four to five amino acids are sufficient to bind IgE and trigger allergic reactions.

PeptideCutter has previously been employed to evaluate the number of pepsin cleavage sites in allergenic proteins (β-casein, β-lactoglobulin, α-lactalbumin, ovalbumin, lysozyme, 7S globulin, Ara h 1, and peach lipid transfer protein) as well as in non-allergenic proteins (horse heart cytochrome c and maize seed storage protein α-zein). The results demonstrated that the non-allergenic maize seed storage protein α-zein was more susceptible to pepsin digestion than the allergenic proteins under both pH 1.3 and 2.0 conditions. Specifically, α-zein exhibited 70 cleavage sites (>0.3 cleavage sites per amino acid), whereas the peach allergen Pru p 3 showed the lowest digestibility, with only 0.09 cleavage sites per amino acid. However, cleavage site simulation results do not always correlate directly with allergenicity. For instance, horse heart cytochrome c—a nonallergenic protein—displayed fewer cleavage sites than several known allergens, including peanut (Ara h 1), cow’s milk (β-lactoglobulin, α-lactalbumin), and chicken egg white (ovalbumin). These findings suggest that while protease susceptibility may provide useful insights into protein stability and potential immunogenicity, it should be interpreted alongside other allergenicity assessment methods for a more comprehensive safety evaluation [70].

### 3.2. In Vitro Approach in Sweet Protein Allergenicity Assessment

#### 3.2.1. Cell-Based Methods

Cell-based allergenicity testing for a protein is based on the mechanism of food allergic reactions. In atopic individuals, upon initial exposure of the immune system to an antigen (sensitization phase), it activates T helper type 2 (Th2) cells instead of Th1 cells. Th2 cells release interleukin-4 (IL-4), which triggers B cells to switch from producing IgG to producing allergen-specific IgE antibodies. After sensitization, repeated exposure to the same allergen strengthens the allergic response by increasing allergen-specific T cell activation and IgE production. These IgE antibodies bind to IgE receptors (FcεRI) on mast cells and basophils, preparing them to react. When the same allergen enters the body again, it binds to the IgE antibodies and causes cross-linking of FcεRI receptors, activating the mast cells. This leads to an immediate allergic reaction. Activated mast cells then release chemical substances such as histamine, IL-4, and other mediators through degranulation, triggering type I hypersensitivity. Other inflammatory cells, including eosinophils and basophils, are involved in the late phase and chronic inflammation [34,72].

Based on the above mechanism, the increase in the allergic mediator (histamine, leukotriene, or β-hexosaminidase) response can be measured, thus evaluating its potential as an allergen. Several cell models that have been used to evaluate allergenic potential include murine peritoneal mast cells, human basophils, rat basophilic leukemia (RBL) cells, and humanized RBL cells [35]. Findings from a systematic literature review indicate the use of rat mast cells and rat basophilic leukemia (RBL-2H3) cells for in vitro allergenicity evaluation of thaumatin and brazzein. Mast cells are employed due to their capacity to undergo degranulation and release mediators upon allergen activation. To investigate mast cell function, RBL-2H3 cells serve as a representative model of human mucosal mast cells. Alongside histamine, these cells release β-hexosaminidase, which can be quantitatively and reliably measured using a spectrophotometric method [73].

In addition to measuring released mediators, mast cell degranulation can also be indirectly observed using microscopy. This approach was applied in studies involving brazzein and monellin, where a suspension of mast cells was incubated with model animal serum and sweet proteins. The number of degranulated cells per 100 mast cells was then quantified under a microscope [24]. However, the specific type of microscope used to assess mast cell degranulation was not reported in the article. According to Yokawa et al. [74], fluorescence microscopy can be employed to visualize the degranulation process by tracking the disappearance of granules within mast cells. This technique enables direct observation of structural changes during mediator release. Other microscopy methods that may be used to evaluate mast cell degranulation include confocal laser scanning microscopy (CLSM) and atomic force microscopy (AFM) [75,76].

Another in vitro method used to evaluate the allergenicity of sweet proteins is the basophil activation test (BAT), which has been applied to thaumatin. This assay utilizes flow cytometry to measure basophil activation induced by allergens in the context of IgE-mediated food allergic reactions. BAT enables the assessment of basophil degranulation levels, which correlate with histamine release [77,78]. Basophil activation can be detected using the CD63 marker, which reflects degranulation. A significant increase in CD63 expression indicates activated basophils [79].

Cell-based models are not only useful for measuring mediator responses in allergy but can also be applied to measure the expression of allergy-related genes. In atopic individuals, the first exposure of the immune system to an antigen (sensitization phase) often results in increased IgE production. This is mainly due to a shift in T-cell differentiation toward the Th2 cell, which promotes the release of IL-4 and IL-13. Key genes involved in this pathway include TNF, CCL11, CCL26, ICOS, IFNGR, STAT3, and TYK2. Among these, CCL11, CCL26, ICOS, and TNF enhance Th2 polarization and thereby stimulate IgE synthesis, whereas IFNGR, STAT3, and TYK2 act as negative regulators of Th2 responses. In addition to Th2 bias, elevated IgE levels also arise from B-cell class switching, where IgM-producing cells are redirected to produce IgE. This process is driven by IL-4, IL-13, IL4R, STAT6, and CD40. Conversely, IL21R, STAT3, and TYK2 inhibit this switching mechanism [80].

In addition to cell line models, food allergenicity testing based on gene expression can also be performed using peripheral blood mononuclear cells (PBMCs). PBMCs offer distinct advantages because they comprise multiple immune cell types involved in allergic responses, including monocytes, natural killer (NK) cells, lymphocytes (both T and B cells), and dendritic cells. This cellular diversity increases the likelihood of identifying relevant biomarkers. Upon activation, PBMCs are capable of producing a wide range of cytokines associated with allergic reactions. Moreover, PBMCs have been reported to express genes such as DEFA1, LTF, AQP3, CLC, TLR4, and IL1R2, as well as key biomarkers including IL-4, IL-5, IL-13, and IL-10 [81]. The use of PBMC-based gene expression assays for allergenicity testing has been demonstrated by [82]. Their study reported that this approach can distinguish between strongly allergenic and weakly allergenic legume proteins, using CCL2, CCL7, and RASD2 as biomarkers. Specifically, the expression of CCL2 and CCL7 was found to be higher when PBMCs were exposed to weakly allergenic proteins (e.g., white bean) compared to strongly allergenic proteins (e.g., soybean). In contrast, RASD2 expression was elevated in response to strongly allergenic proteins.

Cell-based methods can also be applied to mimic the food allergy sensitization pathway in humans by using a stepwise in vitro co-culture model that incorporates all major cell types involved in sensitization, namely intestinal epithelial cells, dendritic cells, CD4+ T cells, B cells, and mast cells. In this approach, HT-29 intestinal epithelial cells are first cultured, followed by the addition of dendritic cells derived from monocytes isolated from healthy donor PBMCs and differentiated for six days. Upon exposure to ovalbumin, activated moDCs are co-cultured with naïve T cells from another PBMC donor and subsequently with naïve B cells to observe the process of isotype switching to IgE. The supernatant from B-cell cultures is then used to stimulate primary human mast cells (derived from CD34+ PBMCs) to assess whether degranulation occurs as a result of IgE crosslinking. Each stage of the experiment is analyzed using flow cytometry, ELISA, and the β-hexosaminidase assay [83].

#### 3.2.2. In Vitro Digestibility

Proteins that exhibit resistance to enzymatic digestion and thermal denaturation have been proposed to possess a greater capacity to induce immune responses, including allergic sensitization, and may be more readily absorbed in a potentially immunogenic or toxic form [30,84]. Although poor digestibility does not inherently indicate allergenicity, digestive stability testing remains a valuable tool, as it provides insight into the persistence of protein fragments that may be recognized by the intestinal immune system. Proteins that are extensively hydrolyzed into small fragments (<2.5 kDa) are generally considered to have a substantially reduced potential to induce sensitization or provoke allergic reactions in sensitized individuals [31].

In vitro digestibility assays can mimic the protein digestion process and thus provide information regarding their stability following enzymatic hydrolysis. This method can refer to the standardized static digestion protocol developed by INFOGEST—a global network comprising over 200 researchers from 32 countries specializing in digestion research—which is physiologically based and includes three sequential phases: oral, gastric, and small intestinal digestion. In this protocol, three simulated digestive fluids are used: simulated salivary fluid (SSF), simulated gastric fluid (SGF), and simulated intestinal fluid (SIF) [36].

The initial phase of protein digestion occurs in the stomach, where proteins are subjected to acidic conditions and enzymatic hydrolysis by pepsin. Pepsin is a nonspecific aspartic protease capable of cleaving a wide range of peptide bonds. As the principal proteolytic enzyme in the gastric environment, pepsin exhibits optimal activity in cleaving peptide bonds adjacent to hydrophobic and aromatic amino acids, such as tryptophan, tyrosine, and phenylalanine. Conversely, it is less effective at cleaving bonds involving small aliphatic residues such as valine, alanine, or glycine. Following gastric digestion, protein fragments or peptides enter the intestinal phase, where they are further hydrolyzed by pancreatic enzymes and bile salts. Proteins that are incompletely digested by gastric and intestinal enzymes—either remaining intact or as large fragments—are more likely to be recognized by the immune system as foreign antigens, thereby increasing the risk of IgE-mediated food allergic responses [85,86].

The stability of a test protein against digestive enzymes can be assessed through techniques such as SDS-PAGE band intensity analysis and mass spectrometry. In addition to these methods, data on the protein’s half-life—defined as the duration a protein remains intact before enzymatic degradation—are critical for interpreting digestibility outcomes. Meanwhile, in vitro digestibility assays can yield meaningful data when conducted under well-defined conditions, including: (i) the use of appropriate control proteins (non-allergenic and standardized proteins); (ii) modeling digestive conditions relevant to vulnerable populations (e.g., infants), including the incorporation of bile salts; (iii) repeated measurement of protein degradation to determine half-life and kinetic constants, with sampling at multiple time points to enable kinetic curve modeling; (iv) application of analytical methods that account for all digestion products (intact proteins, large fragments, and small peptides), while considering complex protein structures such as intramolecular disulfide bonds that may contribute to fragment resistance; and (v) when using densitometric analysis of SDS-PAGE gels, molecular weight markers must be included and protein loading should fall within the linear range of staining to ensure valid quantification [31].

Based on a systematic literature review, thaumatin, brazzein, monellin, and miraculin have undergone in vitro digestibility testing. Thaumatin was reported to be readily hydrolyzed following pepsin and pancreatin digestion, as evidenced by SDS-PAGE analysis; however, the amount of sample and sampling time were not specified [15]. Another in vitro digestibility study on thaumatin was conducted to simulate the oral phase using simulated salivary fluid (SSF), followed by the gastric phase with simulated gastric fluid (SGF) and pepsin for a duration of 6 h. The samples used in this study consisted of a mixture of the two thaumatin isoforms, I and II, with 99.4% purity. During the gastric phase, samples were collected at time points of 0.25, 0.5, 0.75, 1, 2, 3, 4, 5, and 6 h for peptide identification using LC-ToF-MS and quantification by LC-MS/MS. Although this study primarily focused on the analysis of bitter-tasting peptides generated during digestion, the in vitro digestibility results, based on four biological replicates, demonstrated that thaumatin was completely digested, yielding 66 distinct peptides identified over the 6 h digestion period [87]. However, to evaluate the potential allergenicity, the peptide dataset obtained must be compared against allergenic epitopes, which can be analyzed using online resources such as the Immune Epitope Database (IEDB) (https://www.iedb.org/) [88].

For brazzein and monellin, although the evaluation was based solely on band intensity observed in SDS-PAGE, the digestion simulation and sample collection were also conducted across multiple time intervals, allowing for the assessment of band stability throughout digestion. Based on band intensity analysis, the results of simulated two-phase digestion indicated that brazzein bands remained visible even after 120 min of intestinal digestion, although a gradual decrease in intensity was observed. Similarly, the in vitro digestibility test of monellin showed a comparable pattern, with band intensity decreasing progressively during the intestinal phase as digestion time increased. In comparison, bovine serum albumin (BSA) exhibited markedly lower enzymatic stability, as indicated by the complete disappearance of its SDS-PAGE band after only 30 min of gastric digestion [17,19]. In addition to band intensity analysis using SDS-PAGE, LC-MS/MS results following the gastric and intestinal digestion phases demonstrated that monellin is readily degraded into very short peptides (6–22 amino acids), and the distribution of peptide lengths changes according to the duration of digestion time [25].

As previously described, a decrease in band intensity may indicate that the protein has low stability against enzymatic digestion, which in turn suggests a lower risk of allergenicity. However, interpretation based solely on band intensity is insufficient to conclusively determine the allergenic potential of a protein. In addition to assessing band intensity following digestion, LC-MS/MS analysis of digestion products can serve as a complementary approach to evaluate allergenicity based on the resulting peptide profiles. Relevant data may include the total number of peptides generated, the distribution of peptide lengths, and the identification of individual peptide sequences. These peptide sequences can then be compared against allergenic protein databases to assess potential allergenicity [89]. LC-MS/MS analysis of in vitro digestibility products has been conducted for the sweet-tasting protein miraculin. However, the digestion simulation was limited to pepsin treatment and did not include the intestinal phase involving pancreatin enzymes. The resulting peptide sequences generated by enzymatic cleavage were subsequently compared with known allergenic protein sequences using the AllergenOnline database [14].

#### 3.2.3. Indirect ELISA

Allergenicity testing using the ELISA method is based on the interaction between the antigen (target protein) and the primary antibody against the antigen of interest [90]. The antibodies used are from sensitized individuals or from those exhibiting cross-reactivity with the tested protein. This requirement represents a key limitation in evaluating the allergenic potential of novel proteins. The sweet protein miraculin has been evaluated for allergenicity using indirect ELISA due to indications of cross-reactivity with peanut allergens based on results obtained from a commercial ELISA kit. Therefore, when applying ELISA methods to assess the allergenicity of other sweet proteins, it is essential to first consider the potential for cross-reactivity between the target protein and known allergenic proteins. As an initial step, bioinformatic analysis can be performed by inputting the target protein sequence into allergen databases or tools such as AllergenOnline to predict possible cross-reactive allergens.

### 3.3. In Vivo Approach in Sweet Protein Allergenicity Assessment

Several animal models can be used to evaluate allergenicity in vivo. Although interspecies differences exist between animals and humans, the use of animal models remains a critical preclinical step prior to initiating human clinical trials. Both rodent and non-rodent models are employed. Rodents, particularly mice and rats, are widely utilized due to their physiological and genetic resemblance to humans, short reproductive cycles, cost-efficiency, and ease of handling. BALB/c mice are commonly selected for allergy research because of their propensity to polarize toward Th2-type immune responses, characteristic of allergic reactions. Non-rodent species such as pigs, dogs, and primates may also be used, offering closer immunological and physiological parallels to humans [37].

In vivo allergenicity testing of thaumatin was conducted through intramuscular, subcutaneous, intradermal, and intravenous injections in animal models. The intramuscular injection response was evaluated based on ileum contraction, whereas the subcutaneous injection—followed by intradermal and intravenous administration—was assessed using the blueing response in a passive cutaneous anaphylaxis (PCA) assay [21]. Intramuscular injection involves administering the putative allergen into a dense muscle region located just beneath the subcutaneous tissue. This site allows for the rapid absorption of larger solution volumes into the bloodstream via muscle fibers [91]. The intramuscular route may also be combined with the Schultz–Dale method. When the putative allergen (in this case, thaumatin) is injected into the ileum muscle, it can trigger mast cell degranulation, subsequently leading to histamine release [92].

The passive cutaneous anaphylaxis (PCA) test is a standard method used to investigate local inflammation mediated by mast cell activation. Following intradermal injection of serum containing anaphylactic antibodies (induced by prior exposure to a putative allergen), an intravenous injection is administered. The PCA reaction is then assessed by measuring the leakage of Evans blue dye from blood vessels, which occurs due to the release of allergic mediators (e.g., histamine) following allergen–antibody interaction [93]. The intensity of the anaphylactic response is determined by quantifying the amount of Evans blue extravasated into the tissue. A stronger blueing response indicates a higher degree of allergenicity [94].

In addition to injecting proteins into model animals, sensitization of test proteins can be done intragastrically by delivering substances directly into the stomach via oral gavage using silicone tubing, or by injecting the material through the abdominal wall with a needle [95]. Intragastric sensitization has been performed on the sweet proteins monellin and brazzein in guinea pig models, which was then combined with observations on skin, conjunctival, and nasal tests after exposure to putative allergens [24].

The nasal cavity is a highly vascular and mast cell-rich organ, similar to the conjunctiva. Therefore, allergic reactions in this area can be used as an indicator of the severity of an allergic reaction. Provocation testing in this area can be a method for pre-diagnosing food allergies before an oral food challenge, as it is safe, sensitive, and specific, with minimal systemic risks. Symptoms of an allergic reaction in the conjunctiva can include redness and swollen eyelids. Symptoms in the nose can include rhinorrhea, nasal congestion, sneezing, and nasal itching [96].

The use of animal models for immunization with sweet proteins has provided important evidence of cross-reactivity. Antonenko’s study [97,98] demonstrated that mice immunized with monellin generated antibodies capable of recognizing thaumatin. Consistently, Hough and Edwardson [94] reported that rabbit antibodies raised against thaumatin exhibited reactivity not only to thaumatin but also to monellin and other sweet-tasting compounds.

In addition to the methods mentioned above, other in vivo allergenicity tests can be performed by observing the activity of an animal model using a camera and measuring changes in body temperature. In rodents, body temperature can be used as an indicator of allergies. After 30 min to 1 h of allergen exposure, the animal model’s body temperature can be observed. Sensitized animal models will experience a decrease in body temperature (0.5–10 °C) compared to non-sensitized animals. However, this method is subject to bias due to the influence of the laboratory environment, stress levels, and animal strain [99].

### 3.4. Clinical Test Approach in Sweet Protein Allergenicity Assessment

Clinical allergenicity testing involves direct exposure of the test protein to human subjects, either through skin prick testing or oral administration (oral food challenge). The skin prick test (SPT) is a reliable and primary method for diagnosing IgE-mediated allergic diseases (Type I hypersensitivity), including those triggered by inhalant, food, or drug allergens. By introducing the allergen into the epidermal layer, it can cross-link with IgE antibodies already bound to receptors on the surface of mast cells. This interaction induces mast cell degranulation, releasing allergic mediators such as histamine, which subsequently leads to the formation of a wheal (raised bump) and flare (redness) on the skin. These visible reactions serve as surrogate markers for sensitization that may also occur in other organs such as the eyes, nose, or lungs. One advantage of SPT is its localized reaction, confined to the site of allergen administration, allowing multiple allergens to be tested simultaneously. A positive SPT result is defined by a wheal with a longest diameter of ≥3 mm [100].

The oral food challenge (OFC) is considered the gold standard for diagnosing food allergies, including both IgE-mediated and non-IgE-mediated reactions. This test involves the controlled administration of the suspected allergenic food to the patient, thereby carrying a risk of eliciting an allergic reaction. Although OFC is the most accurate method for confirming food allergies, it is not always warranted, particularly when the risk of a severe reaction is high or when diagnostic evidence is already compelling. A positive OFC result is typically indicated by the emergence of clear objective signs of an allergic reaction or the consistent recurrence of subjective symptoms [101].

### 3.5. Limitations

The evidence included in this review is subject to several methodological limitations. As assessed using the GRADE tool, not all studies were of high quality. Some exhibited study limitations (e.g., insufficient methodological detail, unspecified sample size), imprecision (e.g., lack of reported replicates), potential publication bias, and indirectness (e.g., allergenicity assessment not directly measured). The review process itself also faced certain limitations. The review protocol was not prospectively registered in PROSPERO, which limits transparency compared to registered reviews.

Despite these limitations, the findings carry important implications. For practice, the review underscores the diversity of methodologies available for allergenicity testing of sweet proteins, offering guidance for laboratories and food industries in selecting appropriate approaches. For policy, the synthesis highlights the need for standardized regulatory frameworks to ensure the safety of novel proteins introduced into food systems. For future research, this systematic review provides a methodological reference for allergenicity testing of underexplored sweet proteins, thereby contributing to the safety data needed for the development and regulatory approval of sweet proteins as food additives.

## 4. Materials and Methods

This systematic review follows the Preferred Reporting Items for Systematic Reviews and Meta-Analyses (PRISMA) 2020 guidelines [102]. The systematic review protocol was not prospectively registered in PROSPERO. The study had already been completed prior to registration.

### 4.1. Aims and Research Questions

This study systematically reviewed the literature on allergenicity of the seven identified sweet-tasting proteins (thaumatin, monellin, miraculin, pentadin, brazzein, mabinlin, and curculin/neoculin), highlighting the evaluation methods applied to determine their potential allergenic effects. The following research questions were explored in this article: (A) What are the methods used to evaluate the allergenicity of the seven sweet proteins? (B) How does the allergenicity of the seven sweet proteins vary according to each method?

### 4.2. Eligibility Criteria

Articles included in this systematic review are: (i) research articles that explained the method for allergenicity assessment among seven sweet proteins, (ii) full-text available, and (iii) written in English. This review excludes any other non-primary research studies (reviews and book chapters). Studies related to the allergenicity assessment of thaumatin-like proteins, as well as studies unrelated to immunological or allergenicity research on the seven sweet proteins, were also excluded. In addition, relevant papers from the reference lists of the included studies and from reviews identified through the literature search were also included.

### 4.3. Information Sources and Search Strategy

The databases used for literature search were Scopus, PubMed, and Wiley Online Library, with keywords: thaumatin AND (allerg* OR immun* OR safe*), monellin AND (allerg* OR immun* OR safe*), miraculin AND (allerg* OR immun* OR safe*), monellin AND (allerg* OR immun* OR safe*), mabinlin AND (allerg* OR immun* OR safe*), (curculin OR neoculin) AND (allerg* OR immun* OR safe*), “sweet protein” AND (allerg* OR immune* OR safe*). The search was conducted across research articles from all subjects, with no restrictions on the year of publication. The last search was carried out on 30 November 2025.

### 4.4. Selection and Data Collection Process

Articles that met the inclusion criteria from the three databases were first subjected to a duplicate removal process. After duplicates were removed, titles and abstracts of all remaining articles were screened. Articles that did not meet the predefined inclusion criteria were excluded. The results of this initial screening were then re-evaluated through full-text assessment. Based on this assessment, studies that did not fulfill the inclusion criteria or were not relevant to the topic of this review were excluded. The screening process was conducted independently by two authors. In cases of disagreement, resolution was achieved through discussion between the authors. Following these two stages of screening, the final set of articles eligible for synthesis was obtained. The processes of data collection, duplicate removal, and screening were carried out using the free reference management software, Zotero 7.

### 4.5. Data Items

Data extracted from each included study were title, author(s), journal, year and country of publication, allergenicity evaluation method (in silico, in vitro, in vivo, or clinical test), characteristics of sample (sweet protein type, sample preparation, dose), allergenicity determination criteria, and results from the studies.

### 4.6. Study Risk of Bias Assessment

Each of the selected studies was subsequently assessed using the GRADE tool, with evaluation criteria encompassing study limitations, inconsistency, indirectness, imprecision, and publication bias [103]. Although GRADE was originally developed for clinical studies, we adapted its criteria to evaluate in silico, in vitro, and in vivo allergenicity studies. Given the diversity of methodologies among the included studies, the GRADE framework was applied to provide a transparent and standardized appraisal across study designs. This choice was made because no standardized tool currently exists for mechanistic or laboratory-based evidence [104], and GRADE offers a systematic approach, though we acknowledge its limitations outside the clinical context. Finally, based on these five criteria, the overall quality of evidence for each article was determined independently by two authors. Any discrepancies in the assessment were resolved through discussion until consensus was reached.

### 4.7. Effect Measures and Synthesis Method

Studies were included in the synthesis if they reported data on the specified outcomes, including sample characteristics (sweet protein type, sample preparation, and dose), allergenicity assessment method and determination criteria, and the allergenicity potential of each sweet protein. A synthesis approach was employed to categorize the retrieved findings based on the allergenicity evaluation method (in silico, in vitro, in vivo, or clinical test). The narrative structure was designed to highlight the various methods used and the allergenicity potential of each sweet protein according to each method. Results of individual studies were tabulated in summary tables, which included allergenicity assessment and outcomes. Tabulation was employed to facilitate comparison across studies and to highlight similarities and differences in allergenicity evaluation methods of each sweet protein.

## 5. Conclusions

Plant-derived proteins with sweet-tasting properties represent promising candidates for development as low-calorie natural sweeteners, as long as their safety has been thoroughly evaluated. Given their proteinaceous nature, allergenicity assessment is a critical component of safety evaluation. This systematic literature review revealed that comprehensive allergenicity testing has not yet been conducted for all seven identified sweet proteins. Thaumatin, brazzein, monellin, and miraculin are the most extensively studied, with in silico (sequence and structural homology) and in vitro (digestibility and cell-based assays) methods being the most commonly employed. In vivo allergenicity testing using animal models has been conducted for thaumatin, brazzein, and monellin, while thaumatin remains the only sweet protein that has undergone clinical testing in humans, including skin prick tests and oral food challenges. Allergenicity assessments for pentadin, mabinlin, and curculin (neoculin) have not yet been reported, highlighting a valuable opportunity for future research.

## Figures and Tables

**Table 1:** Consolidated methods of allergenicity assessment of sweet proteins.

Method		Allergenicity Assessment	Ref.
In silico	Sequence homology	Comparing similarity between the amino-acid sequence of the sweet protein and the amino-acid sequence of the allergenic protein in the online allergen database: overall protein sequence, 80-amino-acid sliding window, 6–8 mer exact match.	[13,14,15,16,17,18,19,20]
		Measuring the homology indicator between the sweet protein and the allergenic protein using the Receiver Operating Characteristic (ROC) curve.	[16]
	Structural homology	Measuring significance scores (RMSD, Z-score, *p*-value, and TM-score) between the structure of the sweet protein and the allergenic protein.	[16]
		Measuring the percentage identity of the 3D epitope between the structure of the sweet protein and the allergenic protein.	[13]
	In silico digestibility	Measuring the number of cleavage sites and peptide fragment sizes from protease digestion using online prediction software.	[17]
In vitro	Cell-based method	Histamine release assay: measuring histamine concentration in a rat’s mast cell after stimulating with a protein target using fluorimetry.	[21]
		β-hexosaminidase release assay: measuring β-hexosaminidase concentration in rat basophilic leukemia cells (RBL-2H3) after stimulating with protein target using spectrophotometry.	[22]
		Basophil activation test: measuring CD63 expression in basophils isolated from the whole blood of allergic individuals after stimulating with a protein target using flow cytometry.	[23]
		Indirect mast cell degranulation: measuring the percentage of degranulated mast cells obtained from guinea pig blood sera after sensitization with the sweet protein by using a microscope.	[24]
	In vitro digestibility	Evaluating protein stability after in vitro enzymatic digestion based on molecular band pattern in SDS PAGE.	[14,15,17,19,25]
	Indirect ELISA	Measuring absorbance values after incubation with sera from allergic patients to detect potential cross-reactivity of allergenic protein with sweet protein.	[16]
In vivo	Injection, the animal model	Intramuscular injection: measuring the minimum dose of sweet protein that causes the ileum contraction response.	[21]
		Intradermal and intravenous: measuring blueing response (passive cutaneous anaphylaxis dilution titration) after injection of sweet protein.	[21]
	Intragastric administration	Observing skin, conjunctival, and nasal responses after sensitization of the sweet protein to each organ.	[24]
Clinical test	Skin prick test	Measuring the diameter of the wheal in the skin after sweet protein exposure.	[21]
	Oral food challenge	Observing humans’ response after sweet protein exposure.	[21]

**Table 2:** Assessment of risk of bias in selected studies according to GRADE criteria.

Author	Study Limitation	Inconsistency	Indirectness	Imprecision	Publication Bias	Overall Quality
Baniulis et al. [13]	√	√	√	√	√	++++
Tafazoli et al. [14]	√	√	√	X	√	+++
Tafazoli et al. [14]	X	√	√	√	√	+++
EFSA FAF [15]	X	√	√	X	X	++
Menéndez-Rey et al. [16]	√	√	√	√	√	++++
Freeman et al. [17]	√	√	√	√	√	++++
Lynch et al. [18]	√	√	√	√	√	++++
Meetro et al. [19]	√	√	√	√	√	++++
EFSA NDA [20]	Unclear	√	√	X	X	++
Higginbotham et al. [21]	√	√	√	√	√	++++
Chung et al. [22]	√	√	X	√	√	+++
Tschannen et al. [23]	√	√	√	√	√	++++
Novik et al. [24]	√	√	√	√	√	++++
Lifshitz et al. [25]	√	√	√	X	√	+++

GRADE factors: √, no serious limitations; X, serious limitations; unclear, unable to rate item based on available information. For overall quality of evidence: ++ low; +++ moderate; ++++ high.

**Table 3:** In silico approach in sweet protein allergenicity assessment.

Sweet Proteins	Input Data	Methods	Tools	Potential Allergenicity Indicator	Results	Ref.
Thaumatin II	Thaumatin II sequence.	Overall protein sequence homology	Allergen database of the Central Science Laboratory (https://fermi.sdaponline.org/). Allergen online database v.7 (http://allergenonline.com). Structural database of allergenic proteins (https://fermi.sdaponline.org).	A sequence identity ≥ 50% across the overall protein sequence is predictive of significant cross-reactivity with known allergens.	Thaumatin II has sequence identity > 50% in the overall protein sequence with kiwifruit (Act c 2), chili pepper (Cap a 1), Japanese cedar (Cry j 3), and tomato (Lyc e NP24).	[13]
		80-amino-acid sliding window alignment	Allergen online database v.7 (http://allergenonline.com).	A sequence identity ≥ 35% across 80 amino acids is predictive of significant cross-reactivity with known allergens.	Thaumatin II has sequence identity ≥ 35% in 80 amino acids with kiwi fruit (Act c 2), Arizona cypress (Cup a 3), Mediterranean cypress (Cup s 3), mountain cedar (Jun a 3), temple juniper (Jun r 3), eastern red cedar (Jun v 3), apple (Mal d 2), and sweet cherry (Pru av 2).	
		Exact 6-mer word match	Structural database of allergenic proteins (https://fermi.sdaponline.org).	The occurrence of short identical matches (≥6 contiguous amino acids) with known allergens might constitute a linear IgE binding epitope.	Three segments (YTVWAA, TVWAAA, and TGDCGG) in Thaumatin-II showed >46% sequence identity to known plant allergens (Act c 2, Cap a 1, Cup a 3, Jun a 3, and Lyc e NP24).	
	Sequence & structure of thaumatin II & homolog TLPs.	Prediction of antigenic segments and assessment of surface exposure	ClustalW v.1.83 (https://www.genome.jp/tools-bin/clustalw). EMBOSS Antigenic server (http://liv.bmc.uu.se/cgi-bin/emboss/antigenic). CCP4mg v.1.1.1 (https://www.ccp4.ac.uk/).	Predicted antigenic regions with antigenic propensity score and surface exposure.	Thaumatin II has 6 regions that are potentially allergenic epitopes: Ser10-Ala16, Gly73-Cys77, Gly123-Asp129, Pro135-Lys139, Ser155-Thr160, and Lys174-Leu185.	
Miraculin	Miraculin sequence.	Full-length sequence alignment	AllergenOnline, Version 19; (http://www.allergenonline.org/).	A sequence identity of 50–70% and E-value < 1 × 10^−7^ in full-length sequence alignment are predictive of significant cross-reactivity with known allergens.	Miraculin had sequence similarity (26.6–33.5%) with known allergens, including proteinase and aspartic protease inhibitors from *Solanum tuberosum* (potato) and trypsin inhibitors from *Glycine max* (soybean), with reported E-values ranging from 6.4 × 10^−7^ to 6.4 × 10^−10^.	[14]
			BLAST NCBI database.	Percentage identity between miraculin and widely known protein allergens > 50% and high query cover are predictive of significant cross-reactivity with known allergens.	Miraculin sequence has >50% sequence identity with peach, sesame, bitter lemon (Kunitz trypsin inhibitor 2), tomato (miraculin precursor), latex, soy, and peanut, but with low query cover.	[20]
		8-amino-acid sequence alignment	AllergenOnline, Version 19; (http://www.allergenonline.org/).	A sequence identity ≥ 35% across 80 amino acids is predictive of significant cross-reactivity with known allergens.	Miraculin had 7 “80 amino acid” matches (identity > 35%) towards allergenic proteins from *G. max* and *S. tuberosum*.	[14]
		8-amino-acid exact match		The occurrence of identical 8 contiguous amino acids with known allergens is predictive of potential cross-reactivity.	Miraculin had no identical 8 contiguous amino acids with any known allergens.	[14]
	Miraculin sequence & peanut allergen epitope sequence.	Epitope sequence alignment	Allerbase allergen database NCBI BLASTp.	The occurrence of 6–8 contiguous amino acids with known allergens might constitute a linear IgE binding epitope.	The miraculin sequence did not contain any complete epitope (6–8 contiguous amino acids) of the peanut allergen.	[16]
	Pepsin-digested peptides of miraculin.	Full-length sequence alignment	AllergenOnline, Version 19; (http://www.allergenonline.org/).	A sequence identity of 50–70% and an E-value < 1 × 10^−7^ in full-length sequence alignment is predictive of significant cross-reactivity with known allergens.	Peptides from miraculin after pepsin digestion have sequence identities (36–67%) with putative Ani s 11-like protein pr, allergen aspartic protease inhibitor, putative Ani s 11-like protein 2, putative Kunitz trypsin inhibitor, putative trypsin inhibitor, allergen proteinase inhibitor, allergen cysteine proteinase inhibitor, pollen allergen, and putative allergen Pen m 2 Fennero.	[14]
		80-amino-acid sequence alignment		A sequence identity ≥ 35% across 80 amino acids is predictive of significant cross-reactivity with known allergens.	Miraculin has no sequence homology across 80 amino acids to known allergens.	
	Allergens known to exhibit cross-reactivity with peanuts (positive dataset) and those that do not (negative dataset).	Sequence homology based on the Receiver Operating Characteristics (ROC) curve	UniprotKB database (https://www.uniprot.org). AllerBase allergen database (http://bioinfo.unipune.ac.in). NCBI BLASTp (https://blast.ncbi.nlm.nih.gov). GraphPad Prism 6.	Homology indicator (percent identity × percent of coverage) from allergen pairwise sequence alignment.	Miraculin is predicted to have no apparent cross-reactivity with peanut allergen (homology indicator < 0.239).	[16]
Miraculin	Miraculin structure. Peanut allergen (Ara h 1, 2, 3, 5, 8, and 9) structure. Bovine serum albumin (BSA) structure.	Structural homology	Protein Data Bank. UCSF Chimera software (provide RMSD). FATCAT software (provide *p*-value). DALI network server (provide TM-score).	Two proteins present significant structural homology when: RMSD < 2; Z score > 2; *p*-value < 0.001; TM-score 0.5–1.	Miraculin failed to meet any of the four criteria for structural homology with peanut allergens (Ara h 1, 2, 3, 5, 8, and 9).	[16]
Monellin	Monellin sequence.	Full-length sequence alignment	AllergenOnline (Version 21) (http://www.allergenonline.org/). COMprehensive Protein Allergen Resource (COMPARE) (7th iteration) (https://comparedatabase.org/).	A sequence identity > 50% in full-length sequence alignment is predictive of significant cross-reactivity with known allergens.	Monellin has no overall sequence identity > 50% with known allergens.	[17]
		80-amino-acid sequence alignment		A sequence identity ≥ 35% in 80 amino acids is predictive of significant cross-reactivity with known allergens.	Monellin has no sequence identity ≥ 35% in an 80-amino-acid window with known allergens.	
		Amino acid sequences and 3D structural similarity	AllerCatPro 2.0 (https://allercatpro.bii.a-star.edu.sg/).	The percentage identity of a linear 80-amino-acid window and the percentage identity of the 3D epitope are predictive of significant cross-reactivity with known allergens.	Monellin had no evidence of allergenicity (E-value 0.001) and no evidence of similarity to known gluten allergens.	
Monellin	Monellin sequence.	In silico digestibility	Peptide Cutter (https://web.expasy.org/peptide_cutter/).	Number of cleavage sites (more cleavage sites are predicted to be less allergenic).	Monellin had 15 pepsin and 15 trypsin cleavage sites, resulting in an average fragment size of 3.43 amino acids.	[17]
Brazzein	Brazzein sequence.	Full-length amino-acid sequence	AllergenOnline (Version 21; http://www.allergenonline.org/). Allermatch (https://allermatch.org/).	A sequence identity ≥ 70% in full-length sequence alignment, E-value < 0.001, and bit score > 40 are predictive of significant cross-reactivity with known allergens.	From AllergenOnline: Brazzein shared >50% identity with pathogenic-related protein from peach (*Prunus persica*) with E-value 0.76 and bit-score 24.6. From Allermatch: Brazzein shared >50% identity with 20 putative allergens (E-values > 1.1 and bit score < 24.1).	[18]
		80-amino-acid window	AllergenOnline (Version 21; http://www.allergenonline.org/). Allermatch (https://allermatch.org/).	A sequence identity ≥ 35% in 80 amino acids is predictive of significant cross-reactivity with known allergens.	Brazzein shared <35% identity with any known allergens.	[18]
		80-amino-acid window	AllergenOnline database (version 23).	A sequence identity ≥ 35% in 80 amino acids showed significant cross-reactivity with known allergens. E-value < 0.001, bit score > 40.	Brazzein had no identity > 35% with known allergens.	[19]
		8-amino-acid exact match	AllergenOnline (Version 21 & 23; http://www.allergenonline.org/). Allermatch (https://allermatch.org/).	The occurrence of identical 8 contiguous amino acids with known allergens is predictive of potentially cross-reactivity with known allergens.	Brazzein had no 8-amino-acid exact matches with any known allergens.	[18,19]

**Table 4:** In vitro approach in sweet protein allergenicity assessment.

Sweet Proteins	Sample Preparation	In Vitro Model	Treatment	Method	Potential Allergenicity Indicator	Result	Ref.
Thaumatin	Extract of arils, *T. danielli*, from highly selective ultrafiltration	Rat’s mast cell	Sample (thaumatin, various concentrations). Control (Synacthen). Incubation with rat mast cells at 37 °C for 5 min.	Histamine release assay (fluorimetry).	Histamine concentration in the medium.	Thaumatin requires ±1 mM to release 50% of histamine from rats’ mast cells. Synacthen only requires 0.002 mM to achieve the same effect.	[21]
	Thaumatin–gum arabic powder mixture (Naturex, France)	Basophils from whole blood from two allergic (upper airways) symptomatic and four asymptomatic workers	Sample (thaumatin–gum arabic powder 0.01 to 100 µg/mL). Positive control (formyl-methionyl-leucyl-phenylalanine, goat anti-human IgE). Negative control (phosphate-buffered saline). Incubation with whole blood at 37 °C for 15 min.	Basophil activation test (flow cytometry).	High expression of CD63 in basophils.	Two symptomatic workers had basophil stimulation with thaumatin–gum arabic mixture. Four asymptomatic workers had no basophil stimulation with thaumatin–gum arabic mixture.	[23]
	Thaumatin	In vitro protein digestibility	Sample (thaumatin). Comparator (whey protein, egg albumen).	Digestion simulation using pepsin and pancreatin.	Stability to digestive enzyme, as shown by the molecular weight band in SDS PAGE.	Thaumatin was readily hydrolyzed (similar to at least egg albumin or whey protein).	[15]
Brazzein	Recombinant brazzein produced in *Kluyveromyces lactis* strain GG799	Rat basophilic leukemia (RBL-2H3)	Sample (recombinant brazzein 1–15 µM). Positive control (ketotifen fumarate 1–15 µM).	β-hexosaminidase release assay (spectrophotometric).	Release of β-hexosaminidase (absorbance of 405 nm).	Recombinant brazzein (15 μM) inhibited ±27% of β-hexosaminidase release from RBL-2H3 cells.	[22]
	Recombinant brazzein produced in *Pichia pastoris* yeast	Guinea pig blood sera obtained on day 10 after sensitization of brazzein	Sample brazzein: - 2.17 mg/kg body weight. - 21.7 mg/kg body weight. Control (distilled water).	Indirect mast cell degranulation (microscope).	Percentage (number) of degranulated mast cells.	The degranulated mast cell percentage in experimental animals did not significantly (*p* ≥ 0.05) differ from that of control animals.	[24]
Brazzein	Brazzein powder produced from the fermentation of *Komagataella phaffii* (Sweegen)	In vitro digestibility	Sample (brazzein 140 ppm + digestive enzyme). Control (brazzein 140 ppm without digestive enzyme). Positive control (BSA + digestive enzyme).	Gastric phase: 1 mL (140 ppm) of brazzein was digested using pepsin (10 U/µg brazzein) in simulated gastric fluid (SGF) for 2h at pH 2.0. Intestinal phase: brazzein gastric chyme digested using trypsin (3.5 U/μg brazzein) and chymotrypsin (0.04 U/μg brazzein) in simulated intestinal fluid (SIF) for 2 h at pH 6.5.	Stability to digestive enzyme, as shown by the molecular weight band in SDS PAGE.	Brazzein is resistant to gastric (pepsin) enzyme. Brazzein is partially digested by intestinal enzymes.	[19]
Monellin	Recombinant monellin produced in *Pichia pastoris* yeast	Guinea pig blood sera obtained on day 10 after sensitization of monellin	Sample monellin: - 1.45 mg/kg body weight. - 14.5 mg/kg body weight. Control (distilled water).	Indirect mast cell degranulation (microscope).	Percentage (number) of degranulated mast cells.	The degranulated mast cell percentage in experimental animals did not significantly (*p* ≥ 0.05) differ from that of control animals.	[24]
	Serendipity berry (*Dioscoreophyllum cumminsii (Stapf) Diels*) sweet protein produced from the fermentation of *Komagataella phaffii*	In vitro digestibility	Positive control: BSA. Sample: serendipity berry sweet protein containing, by weight, 74.3% monellin and 90% total protein. Negative control: pepsin and pancreatin without serendipity berry sweet protein.	Gastric phase: sample/control was digested using pepsin (10 U/μg sample) in simulated gastric fluid (SGF) at pH 2. Samples were taken at timepoints of 0, 0.5, 1, 2, 5, 10, 20, and 30 min at 37 °C. Intestinal phase: sample from the gastric phase was digested in simulated intestinal fluid (SIF) (pH 7), adding pancreatin, and incubated at 37 °C up to 180 min.	Stability to digestive enzyme, as shown by the molecular weight band in SDS PAGE.	Serendipity berry sweet protein (SBSP) is not resistant to gastric and intestinal enzymatic digestion. SBSP begins to undergo pepsin-mediated digestion after 30 min, and approximately 90% of the protein is fully digested following a two-phase digestion process.	[17]
Monellin	Sweelin (single-chain protein containing DM31), produced through fermentation of a genetically engineered strain of *Komagataella phaffii*	In vitro digestibility	Sample: sweelin (20 or 100 mg of sweelin dissolved in 25 mL of 5 mM sodium citrate buffer at pH 6.0). Positive control: concanavalin A (Con A). Negative control: only digestive enzyme (without protein sample).	Oral phase: sample was digested in simulated salivary fluid (SSF) for 3 min. Gastric phase: sample from oral phase was digested in simulated gastric fluid (SGF) and pepsin (≥3200 U/mg) at pH 1.5. Samples were taken at timepoints of 0, 5, 15, 30, 60, and 120 min at 37 °C. Intestinal phase: sample from the gastric phase was digested in simulated intestinal fluid (SIF) and trypsin (13,000–20,000 BAEE U/mg) and α-chymotrypsin (≥40 U/mg). Samples were taken at timepoints of 0, 5, 15, 30, 60, and 120 min at 37 °C.	Stability to digestive enzymes as shown by the molecular weight band in SDS PAGE. LC-MS/MS analysis of digested peptides.	Sweelin was readily digestible after gastrointestinal digestion (shown by band intensity reduction in SDS PAGE). After intestinal digestion, the LC-MS/MS analysis result showed that sweelin is readily digested into very short peptides.	[25]
Miraculin	Pulp miracle berries were extracted with NaCl 0.5 M and purified with nickel-immobilized affinity chromatography, dialyzed, and freeze-dried	In vitro digestibility	Sample (miraculin 0.1 mg/mL). Control (without miraculin).	Gastric phase: the sample was digested using pepsin (5.45 U/μg) in simulated gastric fluid (SGF) at pH 2. Samples were taken at timepoints of 0, 20, 40, and 60 min at 37 °C.	Stability to digestive enzyme, as shown by the molecular weight band in SDS PAGE.	Miraculin was completely digested within 20 min in the presence of the SGF + pepsin.	[14]
	Lyophilized miracle berry (*S. dulcificum*) extracted with 0.5 M NaCl (pH 6.8), filtered (0.45 µm), and diluted in PBS	Sera from peanut-allergic patients (4) & non-allergic individuals (3)	Sample (miracle berry extract). Positive control (peanut protein extract). Negative control (BSA). Incubated with sera for 3 h at room temperature.	Indirect ELISA.	Absorbance of 450 nm.	Absorbance from miracle berry extract was not significantly different from the negative control in all the analyzed sera.	[16]

**Table 5:** In vivo approach in sweet protein allergenicity assessment.

Sweet Proteins	Sample Preparation	In Vivo Model	Treatment	Method	Allergenicity Indicator	Result	References
Thaumatin	Extract of arils *T. danielli* from highly selective ultrafiltration	Guinea pigs (5)	Thaumatin (50 mg in 0.1 mL) + Freunds Complete Adjuvant/1.3% alhydrogel. Egg albumen (50 mg in 0.1 mL) + Freunds Complete Adjuvant/1.3% alhydrogel.	Intramuscular injection of thaumatin or egg albumen (50 mg/0.1 mL); challenged ex vivo with a range of doses of protein (ileum contraction assay).	Schultz–Dale (ileum contraction response).	Thaumatin (minimum dose 250 ng) can cause an ileum contraction response, which is similar to egg albumen.	[21]
		Rats (5 male)	Thaumatin (10 µg protein) + Freund’s Complete Adjuvant. Egg albumen (10 µg protein) + Freund’s Complete Adjuvant.	First step (Sensitisation): Subcutaneous injection of protein at four separate sites in rats. Second step (PCA analysis): After 11–12 days, animals were killed, and their serum (0.1 mL) was administered intradermally to other rats. Subsequently, after 48 h, animals were challenged with an intravenous injection of protein (2 mg) + Evans blue dye (2%).	Passive cutaneous anaphylaxis (PCA) dilution titration (blueing response).	The PCA titer of Thaumatin is lower than egg albumen (thaumatin: 1 in 8 to 1 in 32, while albumen: 1 in 32 to 1 in 128).	[21]
		Baboons	Sample (thaumatin, various concentrations) 0.1 mL. Negative control (isotonic saline). Positive control (synacthen).	Intradermal injection of thaumatin to the shaved abdomen after an intravenous injection of the indicator dye.	The area of blueing.	Thaumatin (1 mM) provoked a weak response, with blueing area of 0.19 mm^2^, while Synacthen gave a measurable response at a concentration of 0.001 mM.	[21]
Monellin	Recombinant monellin produced in *Pichia pastoris* yeast	Guinea pigs (12 albino males)	Sample monellin: - 1.45 mg/kg body weight. - 14.5 mg/kg body weight.	First step (sensitization): intragastric administration of monellin/brazzein every day for 21 days. Second step (allergy test): after 10–12 days of sensitization, animals were tested: Skin test: a small amount of the sample was applied to the side trimmed (2 × 2 cm) on the animal’s skin surface. Conjunctival test: a drop of the sample solution was pipetted into the conjunctival sac. As control, 1 drop of 0.9% sodium chloride solution is used. Nasal test: a sample solution was pipetted into the nasal passages of animals.	Skin test: Visual examination after 15 min, 30 min, 24 h, and 48 h, presence of hyperemia, presence of infiltration (skin fold thickness). Conjunctival test: vascular pattern of the eye conjunctiva and presence of hyperemia after 15 min, 30 min, and 24 h. Nasal test: hyperemia, sneezing, edema, or increased mucus secretion after 15 min, 30 min, and 24 h.	Skin test: no skin hypersensitivity reaction after 15 min, 30 min, 24 h, and 48 h, absence of hyperemia, absence of infiltration (skin fold thickness). Conjunctival test: no changes in the vascular pattern of the eye conjunctiva and no general hyperemia after 15 min, 30 min, and 24 h. Nasal test: no mucosal hyperemia, sneezing, edema, or increased mucus secretion after 15 or 30 min or 24 h.	[24]
Brazzein	Recombinant brazzein produced in *Pichia pastoris* yeast	Guinea pigs (12 albino males)	Sample brazzein: - 2.17 mg/kg body weight. - 21.7 mg/kg body weight.				

**Table 6:** Clinical approach in sweet protein allergenicity assessment.

Sweet Proteins	Sample Preparation	Subject	Treatment	Method	Allergenicity Indicator	Result	References
Thaumatin	Commercial thaumatin. Purified thaumatin I & thaumatin II as separate solutions. Mixture of thaumatin and arabic gum 1:9 *w*/*w* (freeze-dried). Non-thaumatin plant constituent. Mixture of thaumatin and gum arabic 1:9 *w*/*w* (spray dried).	Human (140 laboratory and pilot plant staff that had been exposed to thaumatin for 7 years)	0.59 mg/mL equivalent to 10.000 pnu/mL for Thaumatin I and II	Skin prick test.	Diameter of wheal.	A total of 67 subjects were allergic to common inhalant allergens. In total, 13 subjects had a positive response to thaumatin (12 subjects were atopic/allergic). Nine of 13 subjects had a positive response to a mixture of thaumatin and gum arabic (addition of gum arabic cannot reduce the allergenicity of thaumatin). Non-thaumatin plant constituent did not cause an allergic reaction.	[21]
	Thaumatin in a gelatin capsule. Lactose in gelatin capsule (as placebo).	Human (10 volunteers; 6 male 4 female)	100 mg/day for each sample in gelatin capsules for 14 days (cross-over for another 14 days)	Oral administration for 14 days for each sample. Prick test before and after the study. Blood IgE and IgG antibody analysis after a 28-day study period. Passive cutaneous anaphylaxis (PCA) test in baboons or rhesus monkeys using serum from each donor.	Skin prick test result. IgE antibodies. Anaphylactic response from PCA test.	No participants showed sensitization to thaumatin after oral consumption. No specific IgE antibodies to thaumatin were detected in participants’ blood. PCA test in baboons or rhesus monkeys showed no anaphylactic antibodies to thaumatin.	[21]
Thaumatin	Mixture of 10% thaumatin and 90% gum arabic (powder form).	Human (8 male employees of chewing gum production plant)	4 symptomatic workers (rhinorrhea, nasal obstruction, and sneezing) had a strong positive reaction to thaumatin	Skin prick test.	Diameter of wheal (qualitative).	Four symptomatic workers (rhinorrhea, nasal obstruction, and sneezing) had a strong positive reaction to thaumatin.	[23]

**Table 7:** Allergenicity potential of sweet proteins assessed based on in silico, in vitro, in vivo, and clinical test methods.

Methods		Allergenicity Potential						
		Thaumatin ^a^	Miraculin ^b^	Monellin ^c^	Brazzein ^d^	Pentadin	Mabinlin	Curculin (Neoculin)
In silico	Sequence homology							
	Structural homology							
	In silico digestibility							
In vitro	Cell-based method							
	In vitro digestibility							
	Indirect ELISA							
In vivo	Injection into the animal model							
	Intragastric administration combined with skin, conjunctival, and nasal test							
Clinical test	Skin prick test							
	Oral food challenge							

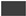
 potential allergenicity observed; 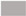 no potential allergenicity observed; 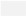 allergenicity assessment not yet conducted; References: ^a^ [13,15,21,23]; ^b^ [14,16,20]; ^c^ [17,24,25]; ^d^ [18,19,22,24].

## Data Availability

The contributions presented in this study are included in the article. Further inquiries can be directed to the author, Nuri Andarwulan.

## Supplementary Materials

The following supporting information can be downloaded at: https://www.mdpi.com/article/10.3390/molecules31091424/s1, Table S1: Articles included in the systematic literature review.

## Abbreviations

The following abbreviations are used in this manuscript:

IDF	International Diabetes Federation
PDB	Protein Data Bank
PRISMA	Preferred Reporting Items for Systematic Reviews and Meta-Analyses
RCSB	Research Collaboratory for Structural Bioinformatics
SCF	Scientific Committee for Food of the European Commission
JECFA	Joint FAO/WHO Expert Committee on Food Additives
FAO/WHO	Food Agricultural Organization/World Health Organization
COMPARE	Comprehensive Protein Allergen Resource
SDAP	Structural Database of Allergenic Proteins
ROC	Receiver Operating Characteristic
PI	Percentage identity
PC	percentage query coverage
BLAST	Basic Local Alignment Search Tools
TLP	Thaumatin-like protein
RMSD	root-mean-square deviation
FATCAT	Flexible structure Alignment by Chaining Aligned fragment pairs allowing Twists
ELISA	Enzyme-Linked Immunosorbent Assays
CD	Cluster of Differentiation
EFSA	European Food Safety Authority
SDS-PAGE	Sodium Dodecyl Sulfate–Polyacrylamide Gel Electrophoresis
LC-MS/MS	Liquid Chromatography-Tandem Mass Spectrometry
PCA	Passive Cutaneous Anaphylaxis
ED	Equivalent Dose
PNU	Protein Nitrogen Unit
OAS	Oral Allergy Syndrome
SGF	Simulated Gastric Fluid
SIF	Simulated Intestinal Fluid
SPT	Skin Prick Test
OFC	Oral Food Challenge

## Appendix A

**Table A1:** Articles excluded from the systematic literature review and the reasons for exclusion.

References	Reason for Exclusion
Hough et al. [97]	Allergenicity studies were not conducted
Van der Wel et al. [105]	Allergenicity studies were not conducted
JECFA [106]	Methods for allergenicity assessment were not explained in detail
McKenzie et al. [107]	Allergenicity studies were not conducted
Cusack et al. [108]	Allergenicity studies were not conducted
Mandal et al. [109]	Allergenicity studies were not conducted
Bodani et al. [110]	Allergenicity studies were not conducted
Reid et al. [111]	Sample used is not sweet protein, Allergenicity studies were not conducted
Antonenko et al. [96]	Allergenicity studies were not conducted
Unterhalt et al. [112]	Full-text unavailable
Unterhalt et al. [113]	Full-text unavailable
Masuda et al. [114]	Allergenicity studies were not conducted
EFSA FEEDAP 2011 [115]	Allergenicity studies were not conducted
EFSA ANS [116]	Allergenicity studies were not conducted
Kukk and Torres [117]	Allergenicity studies were not conducted
Jiang et al. [118]	Allergenicity studies were not conducted
Richter et al. [86]	Allergenicity studies were not conducted
Nakajo et al. [119]	Allergenicity studies were not conducted
Chen et al. [120]	Allergenicity studies were not conducted
Plaza-Diaz et al. [121]	Allergenicity studies were not conducted
Lopez-Plaza et al. [122]	Allergenicity studies were not conducted
Greenaway et al. [123]	Allergenicity studies were not conducted
Álvarez-Mercado et al. [124]	Allergenicity studies were not conducted
Plaza-Diaz et al. [125]	Allergenicity studies were not conducted
Vidal et al. [126]	Allergenicity studies were not conducted
Haimovich et al. [110,127]	Allergenicity studies were not conducted
Bodani et al. [110]	Allergenicity studies were not conducted
Cancelliere et al. [128]	Allergenicity studies were not conducted
Veselovsky et al. [129]	Allergenicity studies were not conducted
Qi et al. [130]	Allergenicity studies were not conducted
Kim et al. [131]	Allergenicity studies were not conducted
Yan et al. [132]	Allergenicity studies were not conducted
Ma et al. [133]	Allergenicity studies were not conducted
Nakajo et al. [134]	Allergenicity studies were not conducted
