# Supplementary material for: Allergenicity Assessment of Plant-Derived Sweet Proteins—In Silico, In Vitro, In Vivo, and Clinical Approach: A Systematic Review
Source: Molecules. 2026 Apr 25;31(9):1424. doi: 10.3390/molecules31091424 (PMC13164861; doi:10.3390/molecules31091424)
Supplement: Supplementary file 1 [file molecules-31-01424-s001.zip › molecules-4209512-supplementary.pdf]

Systematic Review

Allergenicity Assessment of Plant-Derived Sweet Proteins—In Silico, In Vitro, In Vivo, and Clinical Approach: A Systematic Review

Rima Hidayati [1,2], Puspo Edi Giriwono [1,3], Saraswati [1,3], Nuri Andarwulan [1,3,*] and Dominika Średnicka-Tober [1,4,*]

[1] Division of Food Science and Technology, Faculty of Engineering and Technology, IPB University, Kampus IPB Dramaga, Bogor 16680, Indonesia; rimahidayati@apps.ipb.ac.id (R.H.); pegiriwono@apps.ipb.ac.id (P.E.G.); saraswati-sa@apps.ipb.ac.id (S.)
[2] Food Technology Study Program, Asa Indonesia University, East Jakarta 13620, Indonesia
[3] South-East Asia Food and Agricultural Science and Technology (SEAFAST) Center, IPB University, Bogor 16680, Indonesia
[4] Department of Functional and Organic Food, Institute of Human Nutrition Sciences, Warsaw University of Life Sciences, Nowoursynowska 159c, 02-776 Warsaw, Poland
* Correspondence: andarwulan@apps.ipb.ac.id (N.A.); dominika_srednicka-tober@sggw.edu.pl (D.Ś.-T.)

Table S1. Articles included in the systematic literature review.

No.	References
1.	Higginbotham JD, Snodin DJ, Eaton KK, Daniel JW. Safety evaluation of thaumatin (Talin protein). *Food Chem Toxicol.* 1983 Dec;21(6):815-23. doi: 10.1016/0278-6915(83)90218-1.
2.	Baniulis D, Liobikas J, Gelvonauskienė D, Sasnauskas A, Vaitiekaitis G, Stanys V. 2008. Computational analysis of thaumatin-II allergenicity and prediction of antigenic elements of thaumatin-like family proteins. *Biologija.* 54(3):202–207. doi:10.2478/v10054-008-0042-4.
3.	Tschannen MP, Glück U, Bircher AJ, Heijnen I, Pletscher C. 2017. Thaumatin and gum arabic allergy in chewing gum factory workers. *American Journal of Industrial Medicine*. 60(7):664–669. doi:10.1002/AJIM.22729.
4.	EFSA Panel on Food Additives and Flavourings (FAF), Younes M, Aquilina G, Castle L, Engel KH, Fowler P, Frutos Fernandez MJ, Fürst P, Gürtler R, Gundert-Remy U, Husøy T, et al. 2021. Re-evaluation of thaumatin (E 957) as food additive. *EFSA Journal.* 19(11): doi:10.2903/J.EFSA.2021.6884.
5.	Tafazoli S, Vo TD, Roberts A, Rodriguez C, Viñas R, Madonna ME, Chiang YH, Noronha JW, Holguin JC, Ryder JA, et al. 2019. Safety assessment of miraculin using in silico and in vitro digestibility analyses. *Food and Chemical Toxicology* 133. doi:10.1016/j.fct.2019.110762.
6.	EFSA Panel on NDA, Turck D, Castenmiller J, De Henauw S, Hirsch-Ernst KI, Kearney J, et al. Safety of dried fruits of Synsepalum dulcificum as a novel food pursuant to Regulation (EU) 2015/2283. *EFSA Journal* 2021,(6). doi: 10.2903/j.efsa.2021.6600
7.	Menéndez-Rey A, Jerez-Arroyo F, Alegría-Aravena N, Quiroz-Troncoso J, González-Martos R, Sánchez-Díez M, et al. Discarded cross-allergy between miracle berry (Synsepalum dulcificum) and peanut. *Int J Food Sci Technol* 2022, 57(12):7681–90.
8.	Tafazoli S, Vo TD, Roberts A, Rodriguez C, Viñas R, Madonna ME, et al. Corrigendum to "Safety assessment of miraculin using in silico and in vitro digestibility analyses". *Food Chem Toxicol* 2020.
9.	Novik TS, Koveshnikova EI, Kotlobay AA, Sycheva LP, Kurochkina KG, Averina OA, et al. Sweet-Tasting Natural Proteins Brazzein and Monellin: Safe Sugar Substitutes for the Food Industry. *Foods* 2023, 12(22).
10.	Freeman EL, Ward R, Murphy MM, Wang T, Ryder J. Comprehensive safety assessment of serendipity berry sweet pro-tein produced from Komagataella phaffii. *Regul Toxicol Pharmacol* 2024, 147.
11.	Lifshitz Y, Paz S, Saban R, Zuker I, Shmuely H, Gorshkov K, Meetro J, Tafazoli S, Vo T, Amiram G, Levi CS, Lesmes U, Samish I. Safety Evaluation of Serendipity Berry Sweet Protein From Komagataella phaffii. *J Appl Toxicol* 2025, 45(8):1455-1475. doi: 10.1002/jat.4781.
12.	Chung JH, Kong JN, Choi HE, Kong KH. Antioxidant, anti-inflammatory, and anti-allergic activities of the sweet-tasting protein brazzein. *Food Chem* 2018, 267, 163–9.

https://doi.org/10.3390/xxxxx

13. Lynch B, Wang T, Vo T, Tafazoli S, Ryder J. Safety evaluation of oubli fruit sweet protein (brazzein) derived from K om-agataella phaffii, intended for use as a sweetener in food and beverages. *Toxicology Research and Application* 2023, 7.
14. Meetro J, Nahian L, Phipps KR, Vo TD, Dahms I, Lalpuria M, et al. Toxicological Evaluation of the Sweet Protein Brazzein Derived From Komagataella phaffii for Use as a Sweetener in Foods and Beverages. *Journal of Applied Toxicology* 2025, 45(9), 1867–86.
